# Cryo-electron tomography of intact cardiac muscle reveals myosin binding protein-C linking myosin and actin filaments

**DOI:** 10.1007/s10974-023-09647-3

**Published:** 2023-04-28

**Authors:** Xinrui Huang, Iratxe Torre, Michele Chiappi, Zhan Yin, Anupama Vydyanath, Shuangyi Cao, Oliver Raschdorf, Morgan Beeby, Bonnie Quigley, Pieter P. de Tombe, Jun Liu, Edward P. Morris, Pradeep K. Luther

**Affiliations:** 1 Department of Biochemistry and Biophysics, School of Basic Medical Sciences, Peking University, Beijing, 100191 China; 2 Department of Microbial Pathogenesis, Yale School of Medicine, New Haven, CT 06516 USA; 3 National Heart and Lung Institute, Imperial College London, London, SW7 2AZ UK; 4 Thermo Fisher Scientific, Eindhoven, North Brabant Netherlands; 5 Department of Life Sciences, Imperial College London, London, SW7 2AZ UK; 6 Division of Structural Biology, Institute of Cancer Research, London, SW3 6JB UK; 7 School of Molecular Biosciences, University of Glasgow, Garscube Campus, Jarrett Building, 351, Bearsden Road, Glasgow, G61 1QH UK; 8 Department of Physiology and Biophysics, University of Illinois at Chicago, 835 S. Wolcott Ave, Chicago, IL 60612 USA; 9 Phymedexp, Université de Montpellier, Inserm, CNRS, Montpellier, France; 10 Cardiac Function Section, National Heart and Lung Institute, Imperial College London, London, SW7 2AZ UK

**Keywords:** MyBP-C, C-protein, Electron tomography, Cryo-EM, Subtomogram averaging, Muscle regulation, Tokuyasu cryosections

## Abstract

Myosin binding protein C (MyBP-C) is an accessory protein of the thick filament in vertebrate cardiac muscle arranged over 9 stripes of intervals of 430 Å in each half of the A-band in the region called the C-zone. Mutations in cardiac MyBP-C are a leading cause of hypertrophic cardiomyopathy the mechanism of which is unknown. It is a rod-shaped protein composed of 10 or 11 immunoglobulin- or fibronectin-like domains labelled C0 to C10 which binds to the thick filament via its C-terminal region. MyBP-C regulates contraction in a phosphorylation dependent fashion that may be through binding of its N-terminal domains with myosin or actin. Understanding the 3D organisation of MyBP-C in the sarcomere environment may provide new light on its function. We report here the fine structure of MyBP-C in relaxed rat cardiac muscle by cryo-electron tomography and subtomogram averaging of refrozen Tokuyasu cryosections. We find that on average MyBP-C connects via its distal end to actin across a disc perpendicular to the thick filament. The path of MyBP-C suggests that the central domains may interact with myosin heads. Surprisingly MyBP-C at Stripe 4 is different; it has weaker density than the other stripes which could result from a mainly axial or wavy path. Given that the same feature at Stripe 4 can also be found in several mammalian cardiac muscles and in some skeletal muscles, our finding may have broader implication and significance. In the D-zone, we show the first demonstration of myosin crowns arranged on a uniform 143 Å repeat.

**Supplementary Information:**

The online version contains supplementary material available at 10.1007/s10974-023-09647-3.

## Introduction

**Fig. 1:**
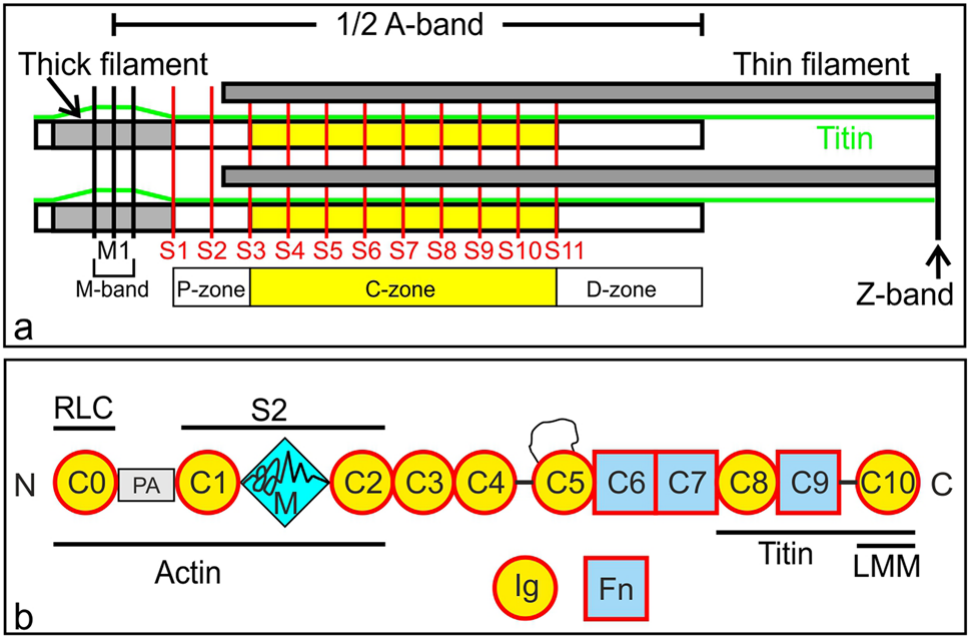
**a** Schematic diagram of a half sarcomere. Thin filaments tethered at the Z-band overlap with thick filaments tethered at their centre at the M-band. Accessory proteins label the thick filaments over 11 stripes, S1 to S11, of spacing 430 Å in each half A-band, grouped in P, C and D-zones. Stripes S3 to S11 mark locations of MyBP-C in C-zones in cardiac and slow skeletal muscle. Long titin molecules attach the Z-band to the M-band and interact through super-repeats with each MyBP-C. **b** Schematic diagram of cardiac MyBP-C comprising Ig- and FN3-like domains C0 to C10, phosphorylatable M-domain between C1 and C2, Pro-Ala linker (PA) between C0 and C1 and a 28-residue loop in domain C5 (Heling et al. 2020). The C-terminal domains C8 to C10 bind titin and myosin LMM backbone. The N-terminal domains bind actin and myosin

Movement is a fundamental characteristic of all animal life. It is brought about by shortening of sarcomeres, the contractile units of muscle, in which arrays of thick myosin filaments slide past overlapping thin actin filaments (Fig. 1a). The motion is triggered by the action of Ca^2+^ on thin filaments causing azimuthal movement of tropomyosin and allowing myosin heads to attach to the thin filaments and cyclically change conformation with hydrolysis of ATP. There is now abundant evidence that the on–off Ca^2+^ switch is not enough to enable the complex range of motions in everyday life (Irving 2017). As in smooth muscle, the thick filament may be involved in the regulation of contraction, and this could be through the protein myosin binding protein C (MyBP-C, C-protein) (Harris 2021; Heling et al. 2020; Irving 2017) (Fig. 1b).

MyBP-C is an accessory protein of the thick filament in vertebrate striated muscle located at 9 of the 11 430 Å-spaced stripes in each half A-band (Fig. 1a). Discovered in the early 70 s (Offer et al. 1973), MyBP-C catapulted to great importance on the discovery of hypertrophic cardiomyopathy (HCM) resulting from mutations in *MYBPC3* (Bonne et al. 1995; Watkins et al. 1995). MyBP-C is a 140 kD rod-shaped protein (Fig. 1b) composed of 10 (skeletal) or 11 (cardiac) immunoglobulin and fibronectin domains of size ~ 40 Å denoted C0 to C10, a MyBP-C specific motif between C1 and C2, the M-domain, and a proline-alanine linker between C0 and C1 (Harris 2021; Heling et al. 2020). The Ig domain C0 is only present in cardiac muscle. Knock-out mice develop HCM (Harris et al. 2002) and mutations in any of the domains can lead to HCM (Carrier et al. 2015; Harris et al. 2011). MyBP-C binds to the filament backbone LMM and titin via C-terminal domains C8 to C10 (Flashman et al. 2007). While the C-terminal domains are associated with the filament backbone, the N-terminal and central domains (C7–C4) and N-terminal domains (C3–C0) are thought to run approximately transversely to the filament from antibody labelling (Lee et al. 2015) with C1 and C0 interacting with the thin filament (Harris et al. 2016). The N-terminal domains C0 to C3 are thought to regulate contraction in a phosphorylation dependant fashion that may be through binding myosin S2/RLC and/or actin (Pfuhl and Gautel 2012; Shaffer et al. 2009). However, the mechanism of the regulation is unknown.

Prior evidence suggests that in resting (diastole) muscle, myosin heads are folded back on the thick filaments in a pseudo-helical fashion in the so-called interacting heads motif (IHM) (Craig and Woodhead 2006; Wendt et al. 2001). This has been equated to the very low metabolic super relaxed state (SRX) (McNamara et al. 2015). Upon activation the myosin heads unfold and this may be aided by phosphorylation of MyBP-C. MyBP-C also binds thin filaments, competing with tropomyosin to activate the thin filaments (Mun et al. 2014). Previs et al. suggested that MyBP-C activation near the centre of the A-band corrects the gradient of the Ca^2+^ activation wave travelling inwards from the Z-band (Previs et al. 2015). The question is how can the N-terminus of MyBP-C interact with both thin and thick filaments? Another related puzzle is how does the thick filament periodicity increase by 1% upon calcium activation of the thin filaments but before the onset on force? (Haselgrove 1975). Irving (Irving 2017) has suggested that MyBP-C connecting actin and myosin, the so-called “C-links”, may provide the answer.

In our previous electron tomography study of fast skeletal muscle, we found MyBP-C formed links with actin filaments (Luther et al. 2011). However, cardiac MyBP-C has distinct differences from the skeletal isoform which include the additional domain C0, 3 phosphorylatable sites in M-domain and a 28 residue loop in domain C5 (Heling et al. 2020). For these differences and for its role in HCM, understanding the structure of cardiac MyBP-C in intact muscle is especially important.

The fundamental requirement for the study of biological structure is the preservation of the desired state. Rapid freezing is now the de facto method to preserve native state in biological samples. Small samples like macromolecules can be applied as a suspension to a grid, blotted to a thin <  ~ 200 nm film and then plunge frozen into a cryogen like liquid ethane at ~ −178 °C. Freezing cellular and thicker samples like muscle is much more difficult. In the 80s and 90s success was achieved on single muscle fibres by slam-freezing against liquid helium cooled metal mirror blocks (Luther et al. 2011; Padron et al. 1988; Tsukita and Yano 1985). No cryo-protectants were used for these studies. The preferred modern method is high pressure freezing often involving some form of cryo-protectant but this method requires considerable effort to achieve success for a particular sample (Studer et al. 2008). After successful freezing, multi-cellular samples like muscle usually need to be thinned down (~ < 200 nm) for examination by electron microscopy. We have previously proposed that an excellent start to high resolution analysis of thicker samples such as muscle fibres is the use of refrozen Tokuyasu-cryosections (Luther and Morris 2003). “Tokuyasu”-cryosections are prepared by chemical fixation, sucrose-cryoprotection, freezing, cutting cryosections and harvesting on grids, followed by thawing, rinsing out the sucrose and contrasting with negative stain (Tokuyasu 1973). However, we (and others) have shown that they can also be studied in the vitreous phase by plunge freezing in liquid ethane and imaged by normal cryo-EM procedures without any heavy-metal stain (Bokstad et al. 2012; Bos et al. 2014; Luther and Morris 2003; Vijayakrishnan et al. 2020). Glutaraldeyde fixation is frequently used in high-resolution cryo-EM studies to stabilise the internal structure (Yang et al. 2020).

Here we have used refrozen Tokuyasu cryosections of rat cardiac muscle to generate cryo-electron tomograms. We find excellent preservation of dimensions, allowing targeting of regions at defined positions from the easily identifiable centre of the M-band. We report here the structure of cMyBP-C at C-zone stripes 3 to 11 derived from sub-tomogram averaging which reveals new details of the linkages with actin filaments previously identified in our studies of skeletal muscle (Luther et al. 2011). We also show that Stripe 4 MyBP-C may be an exception that does not link to actin but may run axially allowing interaction with myosin heads.

## Results

### Cryo-ET imaging of refrozen cryosections of rat cardiac muscle

**Fig. 2:**
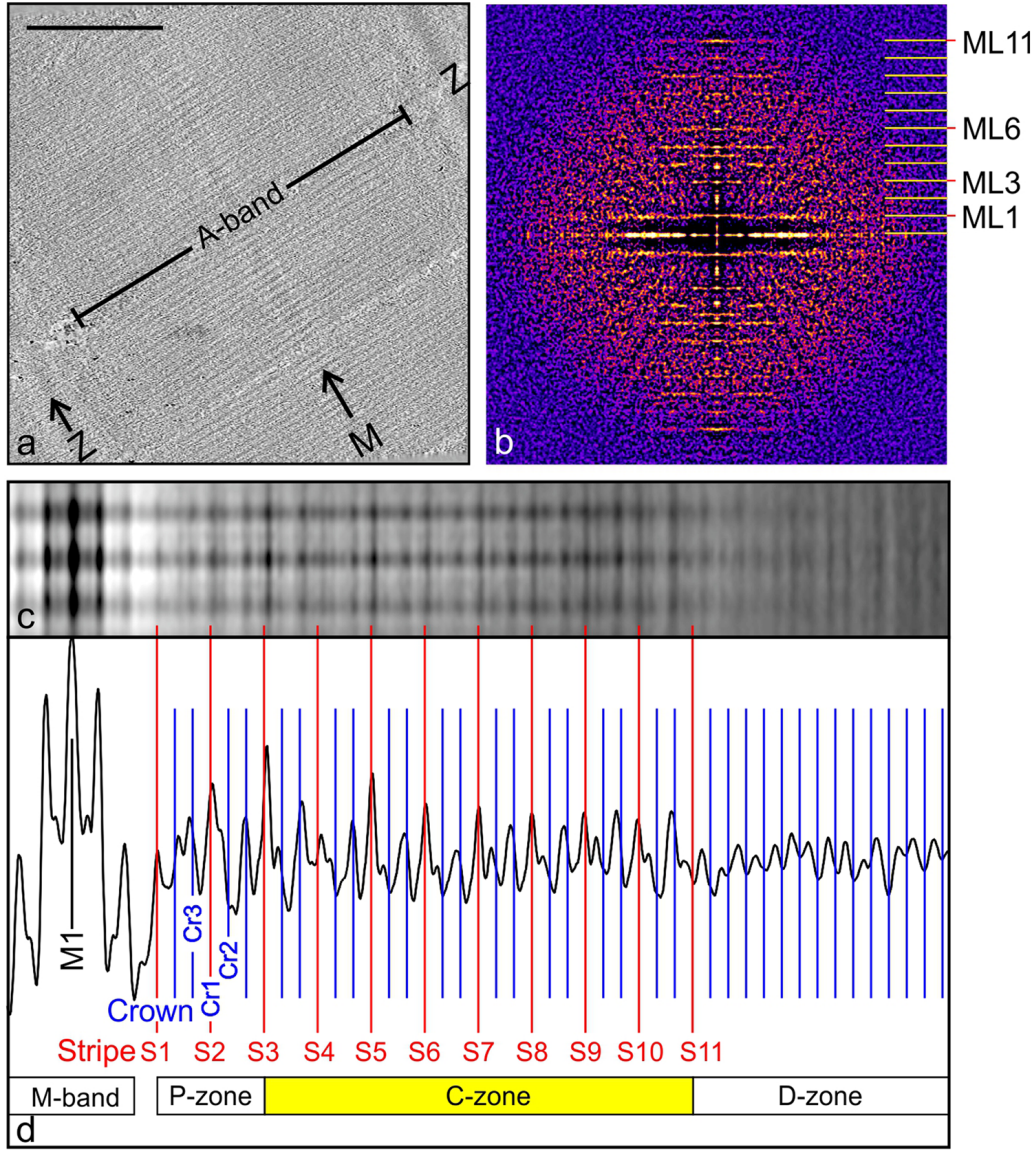
Overview of cryo-EM of rat cardiac refrozen cryosection. Protein density is black in (**a**) and (**c**). (**a**) 2D projected image of a slab of a tomogram (excluding the boundary slices) showing sarcomeres and A-bands running diagonally, Z-bands (Z) and M-bands (M). Scale bar = 0.5 µm. (**b**) Fourier transform summed from all the 10 tomograms used showing clear meridional reflections and meridional layer lines (ML1 etc.) indicating excellent degree of preservation of the helical order of myosin crossbridges. All the myosin layer lines and every 3rd meridional reflection (i.e. 3, 6 etc.) arise from the crossbridge array. The mean transform extends to the 11th order of 430 Å repeat, indicating potential resolution of 39 Å (also see Fig S3, comparison with X-ray diagram). The strong 11th order layer-line corresponding to 39 Å originates from the subrepeat of titin (AL-Khayat et al. 2013). (**c**) Image derived from subtomogram averaging of all the tomograms showing part of the A-band, depicting 3 thick filaments and the M-band at the left. (**d**) Plot profile of mean image (c) aligned precisely below it showing the M-band and central M1 stripe and the approximate 143 Å periodicity of the crossbridge crowns labelled Cr1, Cr2 etc. (blue) found over most of the thick filament. It also shows the accessory protein 430 Å stripes S1 to S11 (red) which coincide with the Crown 1 crossbridges. The C-zone spans stripes S3 to S11 marking the locations of MyBP-C. The D (distal)-zone reveals a constant 143 Å periodicity, which to the best of our knowledge has not previously been observed in vertebrate thick filaments. (Color figure online)

We collected cryo-ET data from the refrozen cryosections of rat cardiac muscle as described in the Materials and Methods (Fig S1 & S2). A representative tilt series is shown in Movie 1 and the corresponding tomogram in Movie 2. A projection image from the same tomogram reveals highly detailed structures (Fig. 2a) including sarcomeres, M-bands (M) and Z-bands (Z). Among 22 tomograms collected, 10 were selected for subsequent subtomogram averaging. The averaged Fourier transform derived from all the selected 10 tomograms (Fig. 2b) showed clear meridional reflections and meridional layer lines of the 430 Å myosin crossbridge repeat (ML1 etc.) extending to a strong 11th order, equivalent to 39 Å, indicating excellent preservation of the approximate helical order of myosin crossbridges in these cryosections. This is also emphasized when we compare the mean Fourier transform to the X-ray pattern of live rat cardiac muscle (Fig S3); the close match reinforces the high quality of our sample preservation. We note that the 11th order meridional reflections has been observed previously and was attributed to LMM packing and the subrepeat of titin domains along the thick filament (AL-Khayat et al. 2013; Kensler 2005).

We first deployed subtomogram averaging of 6x-binned tomograms to analyze a cylindrical volume of 384 pixels in length and 60 pixels in diameter, which includes a central thick filament and neighbouring 6 thick filaments (Fig S4). An average with three thick filaments viewed side-on (Fig. 2c) shows an enhanced banding pattern which is graphically illustrated by the profile plot positioned precisely below (Fig. 2d). The centre of the M-band, M1 provides a prominent landmark for the analysis. The red lines labelled S1–S11 mark the peaks in the plot corresponding to the stripes which mark the location of the non-myosin accessory proteins of periodicity ~ 430 Å, of which stripes 1–3 mark the P-(Proximal) Zone and 3–11 mark the C-zone. Between each pair of stripes are two sub-peaks of approximate periodicity ~ 143 Å arising from the three pairs of myosin heads at each level forming “crowns”. Crown 1 is at the stripe location, Crown 3 is on the M-band side and Crown 2 is on the Z-band side. The D-zone (distal) comprising the remainder of the A-band does not have any prominent stripes, but the plot shows a clear ~ 143 Å periodicity which to our knowledge has not previously been observed in vertebrate thick filaments. We found that the dimensions measured directly on the tomograms using the calibrated magnification of the microscope and the pixel size of the camera agree closely with typical values characteristic of vertebrate cardiac muscle such as the 430 Å repeat of the thick filament. We show in Fig S4 the stepwise determination of C-zone tomograms from 6 × binning to 2 × binning. The analysis reported in this study is from the unbinned tomograms. Table S1 shows the number of subtomograms contributing to the averaged reconstructions of individual stripes and for the class average for all C-zone stripes.

### Subtomogram averaging of individual Stripe MyBP-C 430 Å repeats

**Fig. 3:**
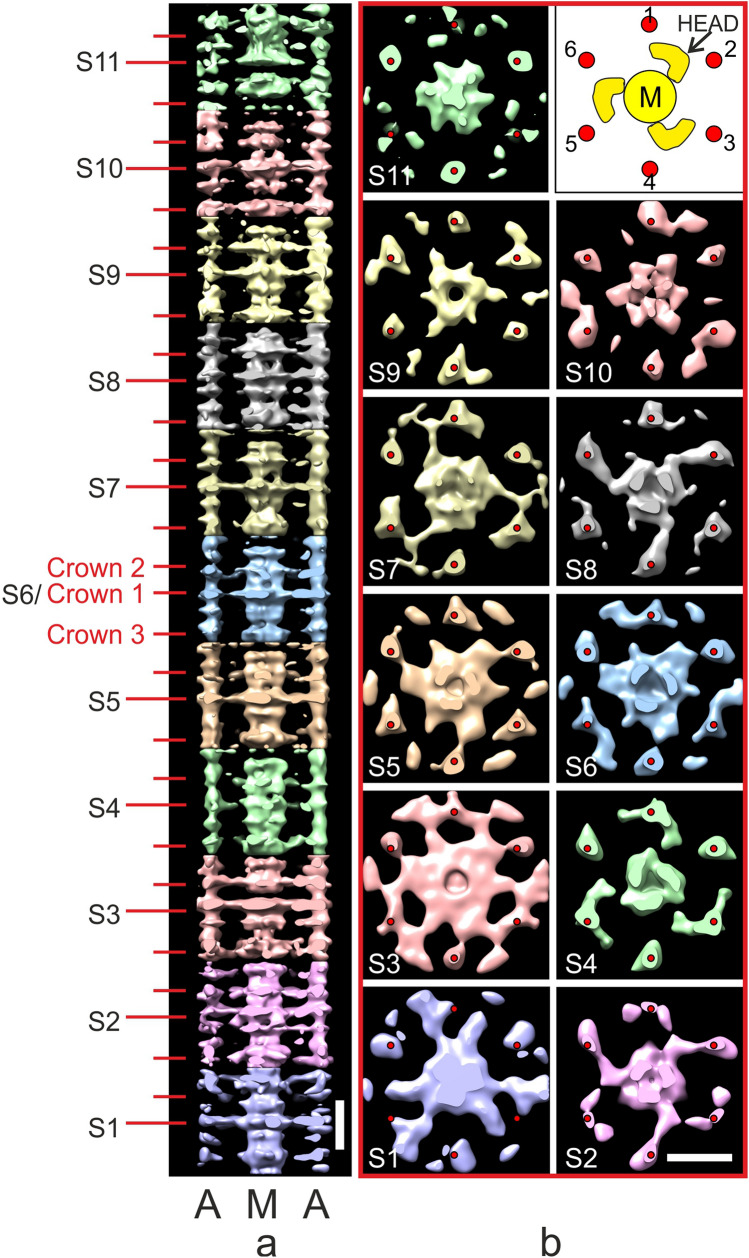
Subtomogram average maps of 430 Å repeats centred at individual Stripes 1 to 11. This figure shows surface rendered views and Fig S5 shows density views. **a** A composite thick filament (M) and two neighbouring actin filaments (A) viewed side-on constructed by stacking axially cropped 430 Å slabs centred at each of the stripes S1 to S11. In each 430 Å band there are 3 crowns of crossbridges, Crown 1 (stripe location), Crown 3 (M-band side) and Crown 2 (Z-band side). The 430 Å C-zone bands have a “perturbed helical” arrangement with Crown separations of 110 Å (Crown 1 to 2) and 160 Å (Crown 3 to 1 and Crown 2 to 3). At each stripe level there is density forming direct links between myosin and actin. (**b**) Cross-section views at the marked stripe levels showing a central myosin filament (M) surrounded by 6 actin filaments as illustrated in the cartoon at top right (red circles, labelled 1 to 6) with outline of myosin heads from Koubassova model (see Fig. 4). At several of the stripe locations (eg Stripe 5, 7 and 8), there is linking density between myosin and actin. Stripe 4 MyBP-C is unusual as discussed in the text and in Figs. 5 and S9 and Movie 4. Stripe 11 shows a compact structure which could indicate close-packed MyBP-C or is the result of higher disorder at the end of the C-zone. Stripes 1 and 2 lack cMyBP-C; nevertheless Stripe 1 shows a link to neighbouring myosin and Stripe 2 shows a link to actin. At the sarcomere length used in this study (2.2 µm), the actin filaments which have length ~ 1 µm (Burgoyne et al. 2008) go past stripe 2 towards the M-band and terminate before stripe 1. The maps at Stripes 4, 5 and 8 are shown as movies 3a, 3b and 3c, respectively. Scale bar = 200 Å. (Color figure online)

A detailed analysis of the MyBP-C structure associated with each of the 11 individual stripes was undertaken using subtomogram averaging of subvolumes made up of individual 430 Å repeats. These subvolumes were centred on the peak of each stripe, comprised a central thick filament and extended laterally to the neighbouring thick filaments corresponding to a region of 583×583×454 Å; Fig. 3 shows the surface rendered view and Fig S5 the density view. Given that the thick filament has threefold symmetry, C3 symmetry was applied to improve the structures (Fig. 3 and Fig S5) and wavelet smoothing applied (Huang et al. 2018). For comparison, we also show the unsymmetrised (C1) version of the cross-sections in Fig S6b in which the views are much noisier.

Figure 3a shows a side-on view of part of a composite thick filament (M) and two thin filaments (A) generated by stacking at 430 Å intervals each of the 11 maps (labelled S1 to S11). The tomogram at each stripe comprises 3 crowns, labelled Crown 1, at the stripe level, Crown 3, on the M-band side and Crown 2, on the Z-band side. We found that the crowns are not equally spaced. This is attributed to perturbations in the thick filament helix in the C-zone (AL-Khayat et al. 2013; Zoghbi et al. 2008). We measured the spacings from the origin points of the heads at each crown and the values were: Crown 1 to Crown 2, 110 Å, and both Crown 2 to Crown 3 and Crown 3 to Crown 1, 160 Å. It is interesting that the ratios of these values, 110:160:160 can be roughly equated to the 3:4:4 ratio derived from the 11 Ig/Fn3 domains in each super-repeat of titin in the C-zone. Values measured in previous studies were a little different as different criteria were used (AL-Khayat et al. 2013; Zoghbi et al. 2008).

Figures 3b shows cross-section views centred at each stripe with applied C3 symmetry. The images show a central myosin filament surrounded by 6 actin filaments (labelled 1 to 6 in cartoon at top right). In some of these cross-section views, there is clear density that appears to form links between myosin and actin e.g. Stripes 3, 5, 7 and 8. As observed previously (Luther et al. 2011), the linking density has a hand at some stripes, eg Stripes 5 and 8, which is predominantly clockwise. At Stripe 7, the density appears to run both ways. Stripe 9 is unusual as the density appears to run straight between neighbouring actins towards a neighbouring myosin filament. MyBP-C at Stripe 4 has much weaker density and is discussed in more detail later. The P-zone Stripes 1 and 2 are devoid of MyBP-C and we do not know the protein content at these stripes. To appreciate the 3D structures in these maps, 3 of the average maps at Stripes 4, 5 and 8 are shown in Movies 3a, 3b and 3c, respectively. The movies show very similar structure in the myosin filament backbone.

### The mean structure of MyBP-C 430 Å repeat

**Fig. 4:**
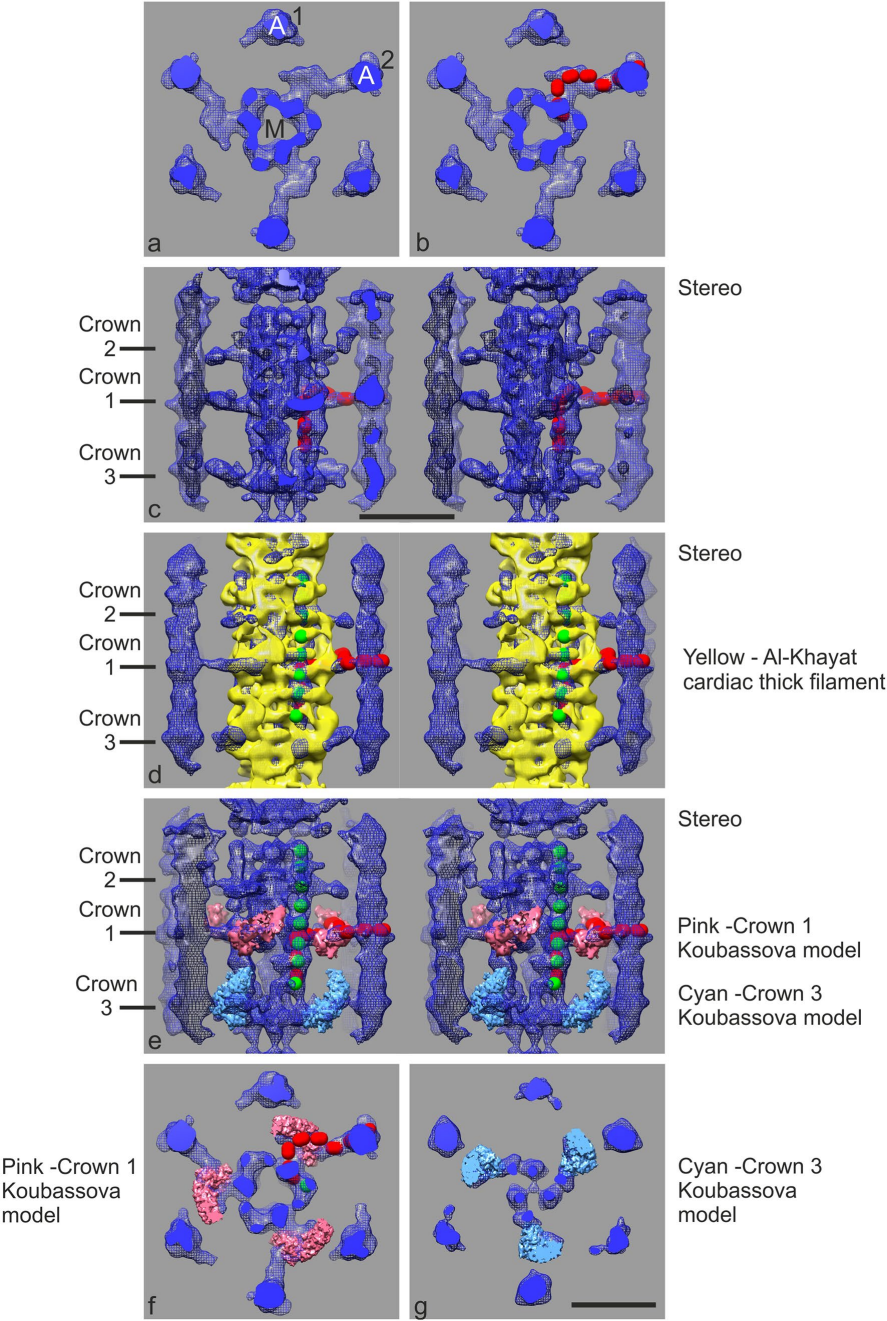
Subtomogram average of all C-zone stripes 3–11. Subtomogram cubes of side 648 Å centred at each stripe were extracted, aligned and averaged and then C3 symmetry applied to produce an average tomogram of a C-zone 430 Å repeat. **a** Thin cross-section slab at MyBP-C stripe showing myosin filament (M) surrounded by 6 actin filaments (A) comprising two sets 1 and 2, due to imposed threefold symmetry. We ascribe the strong density linking myosin to actin set 2 to MyBP-C. **b** fitting along the path of MyBP-C density of 7 40 Å elongated spheres representing Ig-domains. **c** stereo view of side-on view of reconstruction, Crown 3, Crown 1 and Crown 2 mark the position of the myosin crowns; Crown 1 is at MyBP-C stripe position; Crown 3 is 160 Å from Crown 1 on the M-band side and Crown 2 is 110 Å on the Z-band side. The M-band is in the direction of the bottom of the figure. Fitted in the semi-transparent surface are 3 40 Å elongated spheres representing MyBP-C domains 8, 9 and 10 running axially along the myosin filament backbone. **d** Stereo image showing the cardiac thick filament reconstruction of AL-Khayat et al. (2013) in yellow docked into the C-zone tomogram average (blue mesh). Green spheres mark 40 Å periodic density which match the same density identified as titin domains by AL-Khayat et al. (2013 and Zoghbi et al. (2008) and expected from the strong 11th order meridional in the mean Fourier transform (Figs. 2b). **e-g** C-zone tomogram average superimposed with the motor domains of Crowns 1 (brown) and 3 (blue) from the model of (Koubassova et al. 2022), created by removing the light chains and the long S1 α-helices (lever arms); (**e**) Stereo image of side-on view, (**f**) Cross-section view of Crown 1 and (**g**) Crown 3. The Chimera “Fit-in-map” function shows correlation values of the fitting at Crowns 1 and 3 to be 0.53 and 0.62, respectively. These correlation values appear to be consistent with the partial recovery of the myosin head data in the averaged structure. The close coincidence of Crown 1 and MyBP-C suggests that the central domains of MyBP-C can interact with the heads at Crown 1. Scale bar = 200 Å. (Color figure online)

**Fig. 5:**
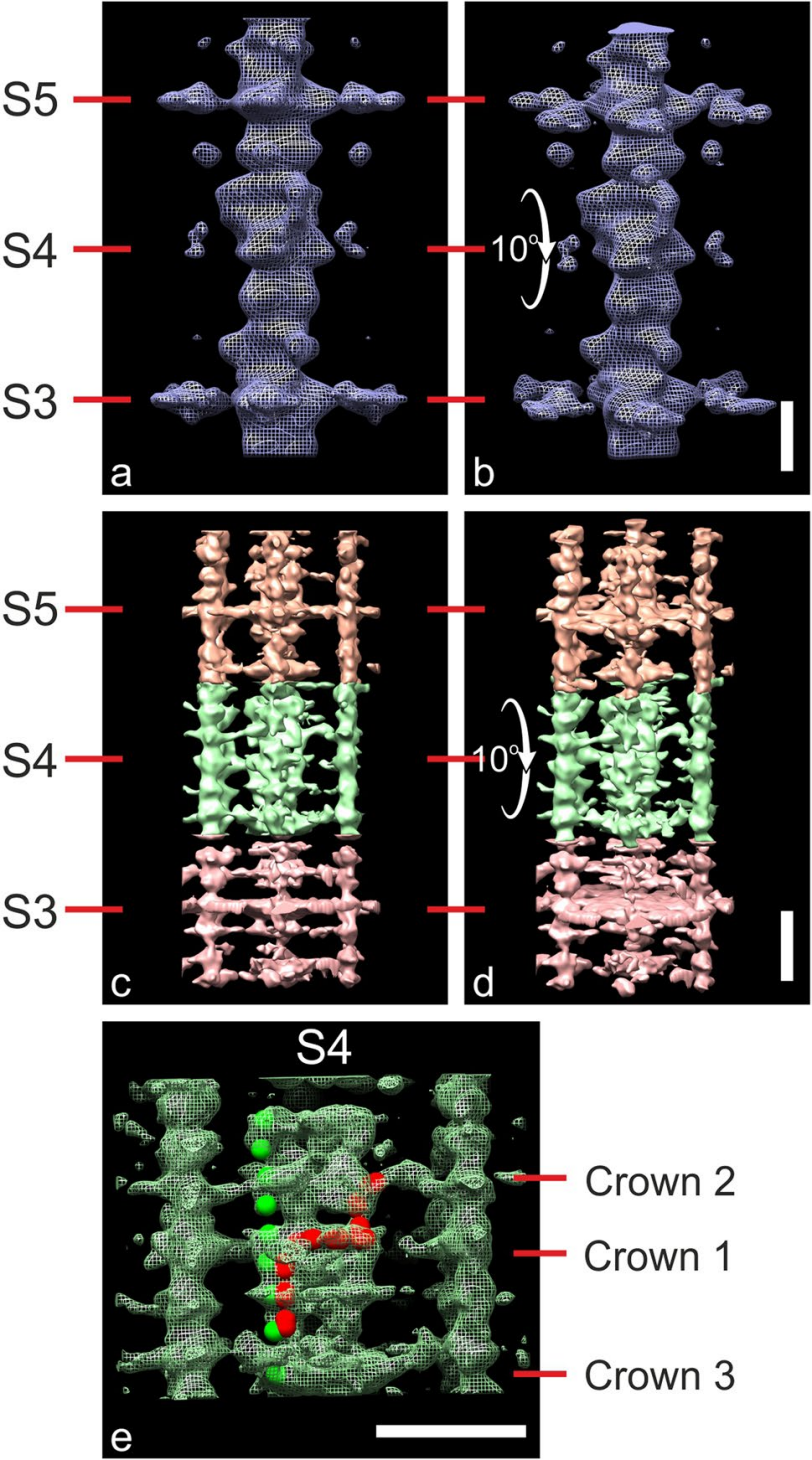
Making the case that MyBP-C at Stripe 4 is unique and may run axially to enable links with myosin head. **a**–**d** Surface rendered views of tomogram segments Stripe 3 to 5 in skeletal muscle (**a**,**b**) derived from earlier study (Luther et al. 2011) and cardiac (**c**,**d**, this study). For both samples, there is distinct lack of radial density at Stripe 4 compared with Stripes 3 and 5. The tomograms in (**b** and **d**) are tilted forward by 10° to reveal “pancake”-like density at Stripes 3 and 5 and lack at Stripe 4. **e** Mesh view of cardiac Stripe 4 with a possible path of MyBP-C fitted with elongated spheres (red). Domains 10–8 have same axial location on myosin backbone as suggested earlier (Fig. 4c, e). The central and N-terminal domains may run axially towards Crown 2. See also Figure S9 showing profile plots of cardiac and skeletal muscles of different species and Movie 4. Scale bar = 200 Å. (Color figure online)

The fine structure associated with the thick filament is more clearly defined after averaging all the C-zone stripes 3 to 11 (Fig. 4) as in previous single-particle studies (AL-Khayat et al. 2013; Zoghbi et al. 2008). The striking feature of the reconstruction is the cross-section structure at the stripe location (Fig. 4a). We see a prominent link between myosin (M) and actin (A) and we ascribe this link to MyBP-C. Broadly equivalent links were also observed in our previous study on subtomogram averaging of frog skeletal muscle in which the sample was rapidly frozen/freeze-substituted and embedded in plastic (see part comparison in Fig. 5) (Luther et al. 2011). However, in the current study these are significantly more clearly defined allowing detailed modelling of MyBP-C. Accordingly, we were able to fit a model made up of 7 40 Å elongated spheres, equivalent to the size of an Ig domain, along the path to simulate MyBP-C domains C7 to C1 (Fig. 4b). We assume that domain C0 attaches further around the actin filament from the C1 domain (Harris et al. 2016). Application of C3 symmetry to the map means that the subunit structure of the actin filament is not recovered as the C3 symmetry does not match that of the actin filament. Nevertheless, the geometry of the attachment of MyBP-C to actin inferred from our map in this way is overall consistent with that observed in studies of the binding of the C0 and C1 domains to actin (Harris et al. 2016; Risi et al. 2018). The side-on stereo view (c) shows the structure of the myosin filaments within a C-zone 430 Å repeat (comprising Crown 3, 1 and 2). Visualisation of the myosin heads is discussed below. Extended density in the backbone allows fitting of 3 40 Å spheres simulating MyBP-C domains C8 to C10 (Fig. 4c) as previously proposed (AL-Khayat et al. 2013; Zoghbi et al. 2008).

We have used C3 symmetry in Figs. 3 and 4 to improve the signal to noise ratio in the regions associated with thick filaments. To more accurately describe the density close to the thin filaments at the expense of some additional noise we present unsymmetrised data in Fig S6. They show that there are 2 or more links made by MyBP-C to actin which are quite compatible with the C3 averaged views in Figs. 3 and 4.

To further understand our structure, we compared it with the previous study by AL-Khayat et al. AL-Khayat et al. (2013) of mammalian (human) cardiac thick filament (Fig. 4d). In Fig. 4d we have docked AL-Khayat’s thick filament on the thick filament in our average map using the “Fit-in-map” tool of UCSF Chimera (Pettersen et al. 2004). Our reconstruction is shown in a blue mesh and AL-Khayat’s filament surface rendered in yellow. The 3 crown levels in the 430 Å repeat are labelled Crown 1 (at MyBP-C Stripe level), Crown 3 (on the M-band side) and Crown 2 (Z-band side). While we here compare with the reconstruction of AL-Khayat et al. we note that the map is very similar to (Zoghbi et al. 2008), hence our comments also apply to Zoghbi et al. Both studies identified periodic density along the length of the filament backbone which they ascribed to titin. We observe a similar periodic density in our reconstruction (shown in green spheres) matching the titin density in AL-Khayat et al. and Zoghbi et al. The periodic density tallies with the strong meridional 11th order (39 Å) reflection we observe in the averaged Fourier transform of the tomogram projections (Fig. 2b). In both studies, the myosin heads in Crowns 1 and 3, were clearer and more prominent compared to Crown 2. A recent study by (Koubassova et al. 2022) suggests that while the myosin filament backbone features in AL-Khayat et al. and Zoghbi et al. are probably correct, the head dispositions in the crowns may be incorrect due to collapse onto the filament backbone as a result of drying of the negative-stained samples. By comparing with cryo-EM imaged filaments of tarantula and scallop, (Koubassova et al. 2022) have proposed a modified vertebrate crossbridge model. We have compared this model to the crossbridge dispositions in Crown 3 and 1 in our tomogram (Fig. 4e–g). We do not compare Crown 2 as it is weak in the tomogram and also in the reconstructions of AL-Khayat and Zoghbi et al. We have also limited the comparison to the motor domains of the model since other regions of the crossbridges are not properly recovered in the subtomogram. Figure 4e shows a stereo view of the side-on view and Fig. 4f and g show cross-section views at Crown 1 and 3 respectively. There is good partial match of the densities. The density at crown 1 in our reconstruction is due to both myosin head and MyBP-C but the limited resolution in the reconstruction (~ 40 Å, Fig S7) does not allow us to distinguish between them. A pertinent feature to note here is that while the N-terminus of MyBP-C is close to actin, the central domains are likely to be closely associated with the myosin heads and hence may interact with them. Fig S8 shows thin density slices through the mean 430 Å MyBP-C region.

### MyBP-C at Stripe 4 is distinctly weak and may have axial orientation

Careful examination of the plot profile of the average ½ A-band in Fig. 2d shows that Stripe 4 is distinctly weak compared to the other MyBP-C stripes 3 to 10 (Stripe 11 is weak probably due to disorder at the edge of the C-zone). On re-examination of the plot profile of our frog skeletal muscle tomography study (Luther et al. 2011) we find the same effect. For ease of comparison, both are reproduced in Fig S9 (a & g). We then prepared 3 new cardiac and 1 skeletal cryosection samples and prepared their plot profiles (Fig S9). We also examined a previously published cardiac (human) plot profile (Fig S9c) (Vydyanath et al. 2012). In total 7 samples were compared, and they all show profile plots with reduced Stripe 4 peaks. We next compared the mean tomograms of rat cardiac (this study) with frog skeletal (Luther et al. 2011). Figure 5 shows segments comprising Stripe 3 to 5 of the skeletal tomogram of Luther et al (2011) (a, b) and the present cardiac study (c, d). At Stripe 4, we see little actin-directed density for both. The difference is enhanced in the 10° tilted forward views (Fig. 5b&d). The cardiac reconstruction shows an almost pancake-like density at Stripes 3 and 5 compared with near absent density at Stripe 4. We note here that frog sartorius muscle is a fast muscle; in such muscles the fast isoform of MyBP-C is present over stripes 5 to 11. However fast muscles also have slow MyBP-C (up to 25% of total MyBP-C) (Geist et al. 2018; Li et al. 2017); slow MyBP-C is present over stripes 3 to 11 (Bennett et al. 1986; Li et al. 2017), hence comparison of C-zone stripes in amphibian skeletal and mammalian cardiac muscle is justified. Since we know that MyBP-C is present at Stripe 4, the weak density could mean that it is floppy or could follow an axial path. We speculate that an axial path is possible in which the central and N-terminal domains run mostly axially towards the heads on Crown 2 and may form the highly sought link of MyBP-C to myosin S2 (Gruen et al. 1999; Pfuhl and Gautel 2012). Figure 5e illustrates one possible path for MyBP-C at Stripe 4 with domains C7 to C5 running circumferentially with the N-terminal domains running axially with potential interaction with myosin head and S2. Such a scheme accounts for the length of MyBP-C. However, a scheme with domains C7 to C0 running axially with part looping off the myosin is also possible. A comparison of the cardiac and skeletal tomograms for Stripe 3, 4 and 5 is shown in Movie 4.

## Discussion

The holy grail of research on cMyBP-C is the determination of the mechanism by which it regulates contraction in striated muscle. The results of our study provide some clues. We have examined relaxed rat cardiac muscle in refrozen Tokuyasu cryosections and carried out cryo-ET and subtomogram averaging of the MyBP-C 430 Å repeats. We find on average MyBP-C binds actin across a disc perpendicular to the thick filament. The visualisation is clearer, better defined and has higher resolution than our previous electron tomography of frog skeletal muscle (Luther et al. 2011). The exception is the weak Stripe 4 which could be due to axial orientation of MyBP-C.

Cardiac MyBP-C is highly phosphorylated under basal conditions (Jacques et al. 2008) but we do not know how it affects the configurations of MyBP-C that we have observed here. We note that our subtomogram average shows MyBP-C emerging from a common density shared with myosin heads but our resolution of about 40 Å (Fig S7) does not distinguish between them. Nevertheless, it is the central domains of MyBP-C, from about C6 to C4, which would be present in this region and being in close proximity to the myosin heads, could be interacting with them. Indeed very recently Ponnam and Kampourakis (2021) have shown that central domains of MyBP-C (C2-C4, C5-C7) bind to the myosin head in a phosphorylation independent fashion.

### Use of Tokuyasu cryosections for cryo-EM studies

We have shown here that the Tokuyasu method involving chemical fixation (Tokuyasu 1973), is a quick and inexpensive method to produce cryosections which upon refreezing can be used for cryo-EM. The close match of the averaged Fourier transform of our tomogram projections with the X-ray pattern of live cardiac muscle (Fig S3) shows impeccable preservation of the fine structure. Prominent layer lines in the mean Fourier transform show the presence of helically arranged crossbridges and the strong 11th order of the 430 Å crossbridge-repeat, equivalent to 39 Å, matched the titin domains observed along the length of the thick filament backbone (Fig. 4). The dimensions were preserved precisely and enabled us to identify each of the 11 stripes at the predicted locations from the centre of the M-band. We note that for general use, researchers would have to assess the effect of chemical fixation on their sample. Nevertheless the ideal method currently for a cellular sample requires rapid freezing, probably using high pressure freezing (Studer et al. 2008) and preparation of frozen lamellae using focussed ion beam (FIB) milling (Mahamid et al. 2016; Wang et al. 2021).

### Individual stripe MyBP-C 430 Å repeats

The subtomogram average maps of the individual stripes (Figs. 3, S5 and S6) show common features in the filament backbone over the 430 Å repeat. There is higher density at each MyBP-C level with varying protrusions towards or links with actin. Since the symmetry and repeats of myosin and actin are different, each of the three MyBP-C proteins at a particular stripe, exploring for target binding sites on actin would have different paths and may or may not form a link with actin. Indeed, in the unsymmetrised maps shown in Fig S6 we see 2 or more MyBP-C links to actin.

We noted different dispositions of MyBP-C at the different stripes. The structure of MyBP-C at Stripe 4 appears unique, and we have shown this by two different methods. Firstly, we compared the plot profiles of five different mammalian cardiac muscles and two skeletal muscles and found that Stripe 4 is consistently weaker than at the other MyBP-C stripes. Secondly, our subtomogram average of Stripe 4 shows hardly any MyBP-C protrusion and this is also the case with our previous skeletal tomography study (Luther et al. 2011). The weakness of Stripe 4 may arise from a floppy or axial disposition of MyBP-C; if axial the central and N-terminal domains may run towards Crown 2. This would make possible contact with myosin S2 of Crown 2 – a much sought after binding partner of MyBP-C (Gruen et al. 1999; Pfuhl and Gautel 2012). We note that Stripe 4 is near the start of the C-zone and at the range of sarcomere lengths during contraction in cardiac muscle, 1.6 to 2.3 µm (de Tombe and ter Keurs 1991), Stripe 4 MyBP-C will always be overlapped by part of thin filaments. As the heads of adjacent crowns have intermolecular interactions (AL-Khayat et al. 2013; Gonzalez-Sola et al. 2014), it is possible for the effect of Stripe 4 to be transmitted along the thick filament. Some previous studies have proposed models of MyBP-C configuration in which the central and N-terminal domains run axially along the filament labelling myosin heads (Kampourakis et al. 2014; Nag et al. 2017). Such axial paths would not form distinct stripes. These models therefore can only apply to Stripe 4 MyBP-C.

### Previous studies of MyBP-C binding to actin and myosin

There is a large body of evidence of MyBP-C N-terminal domains binding actin. This includes centrifugation assays (Moos et al. 1978; Razumova et al. 2006; Shaffer et al. 2009), fluorescence light microscopy (Moos 1981; Rahmanseresht et al. 2021), electron microscopy (Moos et al. 1978),(Kensler et al. 2010; Shaffer et al. 2009), neutron scattering (Whitten et al. 2008), in vitro motility assay (Previs et al. 2012; Razumova et al. 2006; Weith et al. 2012). In the last decade structural evidence has accumulated through single particle EM (Mun et al. 2014) and cryo-EM (Harris et al. 2016; Risi et al. 2018). Ideally, we should fit the cryo-EM structures into our map, however we have not reconstructed thin filaments independently of the thick filaments. There is considerable evidence of the N-terminal domains binding myosin head S1 and S2: biochemical (Gruen and Gautel 1999; Nag et al. 2017) and NMR scattering (Gruen et al. 1999). Here we have found that only at Stripe S4 MyBP-C could bind S2. The conformation proposed by Lee et al. (Lee et al. 2015) comprising MyBP-C looping back to bind myosin head would preserve the stripes but our tomography does not show evidence of this.

We have compared our subtomogram averages of C-zone stripes with the human cardiac thick filament structure derived from isolated filaments (AL-Khayat et al. 2013) and with the model of Koubassova et al. (2022) in which the myosin heads are observed to adopt the IHM structure (Fig. 4). We observed sufficient agreement within the cross-bridge region to assign density to myosin heads within our tomograms with some confidence. However, in our current analysis it is difficult to ascertain whether the myosin heads fully adopt the IHM configuration seen in human cardiac thick filaments or some related arrangement, perhaps due to limiting resolution (~ 40 Å for the C-zone stripe average). Furthermore, some variation in the radial location of myosin head density was observed at different crown levels. In addition, significantly weaker myosin head density is found at Crown 2 compared with Crown 1 and Crown 3. Weaker myosin head density for Crown 2 was also observed in the structural analysis of isolated cardiac thick filaments (AL-Khayat et al. 2013; Zoghbi et al. 2008). Subtomogram averages derived from individual stripes appear to show more extensive variation associated with the myosin head density, but here the noise level makes it difficult to reliably assess the configuration of the myosin heads. These observations may be related to previous tomographic analysis of isolated tarantula thick filaments in which the IHM motif is only revealed infrequently (Marquez et al. 2014).

### Role of MyBP-C in thick filament-based regulation of cardiac contraction

In the last decade, intense research has been done to establish whether regulation of vertebrate striated muscle has a thick filament basis in addition to the established thin filament actin-TM-TN system (Brunello et al. 2020; Heling et al. 2020; Irving 2017). This could be due to MyBP-C forming links to actin and stabilising the conformation of pairs of myosin heads (Woodhead et al. 2005) – the interacting heads motif (IHM) – and promoting quasi-helical order with folded heads in the C-zone. The IHM state is now thought to represent a super relaxed state (SRX) in which ATP turnover is extremely low (Cooke 2011).

A plausible model of thick filament based regulation is based on the ~ 1% increase in the thick filament spacing that occurs ahead of force development upon activation (Haselgrove 1975; Irving 2017; Reconditi et al. 2014). Haselgrove (1975) proposed that the extension could be due to a change in the LMM packing of the backbone of the myosin filaments. We suggest here that the change could be produced by the tension in MyBP-C C-terminus transmitted back from the actin bound N-terminus i.e. through “C-links” (Irving 2017). The curved path of MyBP-C is conducive to such a torque on the thick filament backbone and could cause some unravelling of the helical packing. There could be an accumulative torque from the C-zone links in the other half of the A-band. As suggested by Haselgrove and others (Irving 2017), this change could activate the thick filament, forming a hub of regulation by the thick filaments. In another part of the sarcomere, such a torque effect was found in the morphology of the Z-band in the lattice changes due to change in tension from relaxed to active muscle (Oda and Yanagisawa 2020).

## Conclusions

We have shown in this study that in relaxed mammalian cardiac muscle, MyBP-C on average make direct links between thick and thin filaments. This gives support to models of thick filament-based regulation of contraction in which MyBP-C links upon thin filament activation by calcium immediately affect thick filament backbone packing and activate myosin heads off their close packed helical tracks. We examined Stripe 4 MyBP-C in different muscles and species and found it is different and we infer it may run axially and interact with myosin head or S2 of an adjacent crown. Further work is required to improve the resolution of the interactions and to see the structural changes in different states of activation.

## Materials and methods

Fig S1 outlines the different steps for this study including sample preparation and electron tomography.

### Sample preparation

Rats (Sprague–Dawley strain) were sedated with isofluorane and sacrificed by cervical dislocation in compliance with UK Home Office Schedule 1. Papillary and trabeculae muscles were dissected under Krebs solution with 30 mM 2,3-butanedione monoxime (BDM) added and pinned on Sylgard in a Petri dish. The muscle was aerated for 30 min with 95% oxygen/5% carbon dioxide. The samples were fixed for 1 h with 3% glutaraldehyde in Krebs solution. After rinsing with Krebs, the fixed muscles were immersed in cryoprotectant solution of 2.3 M sucrose in Krebs and left in a cold room overnight. Small, 0.5 to 1-mm-cube pieces were cut, mounted on a cryopin, excess sucrose blotted and the cryopin frozen by plunging into liquid nitrogen. Cryosections ~ 100 nm thick were cut at −100 °C with a Leica UC6 ultramicrotome fitted with a Leica FC6 cryochamber, transferred to formvar/carbon coated nickel grids, floated on PBS buffer in well-plates to rinse off the sucrose in a fridge for ~ 1 day.

### Freezing

Prior to freezing, 10 nm gold fiducial suspension was applied for 1 min. The gold suspension was made following the method of Slot and Geuze (1985). The grids were frozen using a Vitrobot Mark IV and stored in liquid nitrogen.

### Cryo-electron tomography and sub-tomogram averaging

Cryo-ET imaging was performed at eBIC laboratory at Diamond Light Source, Didcot, with a Titan Krios operated at 300 kV and a Gatan K2xp direct detector. Ten grids were loaded into the Krios auto-loader and the best grid was selected. Fig S2a shows part of the atlas image with myofibrils running diagonally to the upper left. ~ 2 µm square regions were selected for the tilt-series (Fig S2b). Images were acquired at × 26,000 magnification in movie mode from −60° to 60° with 3° steps using SerialEM (Mastronarde 2005). The pixel size was 5.4 Å and image size 4 K × 4 K. The total dose for a complete tilt series was 100 e/Å^2^. Frames were aligned with the MotionCorr algorithm (Li et al. 2013), or with the frame alignment algorithm built into SerialEM and aligned frames were stacked into tilt-series. The tilt-series were automatically aligned using fiducial alignment with “Batchruntomo” script and then manually checked to reduce the alignment error with etomo in IMOD (Kremer et al. 1996). The tilt-series with poor fiducial alignment were discarded. Poor fiducial alignment was defined as alignment residual above 1 pixel or retaining fewer than 20 tilt images. 18 tilt-series were selected from 20 for further processing. Defocus for each tilt in the aligned tilt series was determined by GCTF (Zhang 2016). Tilted images were CTF-corrected using ctfphaseflip in IMOD (Xiong et al. 2009). Tomograms were reconstructed using weighted back projection in Tomo3D (Agulleiro and Fernandez 2015). Tomograms with distorted myosin filaments were discarded and finally 10 tomograms were selected.

The 10 tilt-series were binned 6x (32.4 Å/pixel), 4x (21.6 Å/pixel) and 2x (10.8 Å/pixel) to reconstruct their tomograms. CTF-corrected unbinned tomograms were reconstructed to obtain the highest possible resolution in filament segments of interest. The subtomogram averaging for filament segments of interest was performed with Dynamo (Castano-Diez et al. 2012). All the tomograms were first rotated to make Z-axis along the filaments to comply with the coordinate convention in Dynamo and to make it easier to pick and extract the filament segments (Fig S4a). Subtomogram averaging with stepwise reduction in binning starting from 6x, was done as shown in Figure S4. First, using a template created from one tomogram (Fig S4bb), a 6 × binned average map based on M1-centre was generated. This was then further refined to 4 × binned map first centred on M1 (Fig S4d) and then centred on stripe 7 with a smaller box of 384 pixels for each C-zone (up and down, Fig S4e). This was repeated with a smaller box of 240 pixels covering the stripe zones (Fig S4f). Finally, the up and down stripe zones were combined to produce a 4 × binned average (Fig S4g). Cross-sections shown below each side-on view show increasingly improved detail from (b) to (g).

The averaged Fourier transform (Figs 2b, S3b), was calculated from the 10 tomograms by projecting each tomogram along its Z-axis to create a set of 2D images. These images were then rotated to align the thick filaments with the y-coordinate axis and divided into individual A-bands. Fourier transforms were calculated from each of the half A-band images and their amplitude spectra were summed to calculate an averaged Fourier transform. This was then averaged with its mirror image to produce the final average.

### Supplementary Information

Below is the link to the electronic supplementary material.

Supplementary file1 (DOCX 3622 KB)

Supplementary file2–Movie 1 (M4V 14195 KB)

Supplementary file3–Movie 2 (M4V 6661 KB)

Supplementary file 4–Movie 3a (MP4 3237 KB)

Supplementary file 5–Movies 3b (MP4 2728 KB)

Supplementary file 6–Movies 3c (MP4 3291 KB)

Supplementary file 7–Movie 4 (M4V 20610 KB)

## Data Availability

The C3 mean structure of cardiac MyBP-C 430 Å repeat has been deposited to the Electron Microscopy Data Bank, accession code EMD-14504

## Abbreviations

MyBP-C, C-protein: Myosin binding protein-C
TM: Tropomyosin
TN: Troponin

## References

[CR1] Agulleiro JI, Fernandez JJ (2015). Tomo3D 2.0–exploitation of advanced vector extensions (AVX) for 3D reconstruction. J Struct Biol.

[CR2] Al-Khayat HA, Kensler RW, Squire JM, Marston SB, Morris EP (2013). Atomic model of the human cardiac muscle myosin filament. Proc Natl Acad Sci U S A.

[CR3] Bennett P, Craig R, Starr R, Offer G (1986). The ultrastructural location of C-protein, X-protein and H-protein in rabbit muscle. J Muscle Res Cell Motil.

[CR4] Bokstad M, Sabanay H, Dahan I, Geiger B, Medalia O (2012). Reconstructing adhesion structures in tissues by cryo-electron tomography of vitrified frozen sections. J Struct Biol.

[CR5] Bonne G, Carrier L, Bercovici J, Cruaud C, Richard P, Hainque B, Gautel M, Labeit S, James M, Beckmann J (1995). Cardiac myosin binding protein-C gene splice acceptor site mutation is associated with familial hypertrophic cardiomyopathy. Nat Genet.

[CR6] Bos E, Hussaarts L, van Weering JR, Ellisman MH, de Wit H, Koster AJ (2014). Vitrification of Tokuyasu-style immuno-labelled sections for correlative cryo light microscopy and cryo electron tomography. J Struct Biol.

[CR7] Brunello E, Fusi L, Ghisleni A, Park-Holohan SJ, Ovejero JG, Narayanan T, Irving M (2020). Myosin filament-based regulation of the dynamics of contraction in heart muscle. Proc Natl Acad Sci U S A.

[CR8] Burgoyne T, Muhamad F, Luther PK (2008). Visualization of cardiac muscle thin filaments and measurement of their lengths by electron tomography. Cardiovasc Res.

[CR9] Carrier L, Mearini G, Stathopoulou K, Cuello F (2015). Cardiac myosin-binding protein C (MYBPC3) in cardiac pathophysiology. Gene.

[CR10] Castano-Diez D, Kudryashev M, Arheit M, Stahlberg H (2012). Dynamo: a flexible, user-friendly development tool for subtomogram averaging of cryo-EM data in high-performance computing environments. J Struct Biol.

[CR11] Cooke R (2011). The role of the myosin ATPase activity in adaptive thermogenesis by skeletal muscle. Biophys Rev.

[CR12] Craig R, Woodhead JL (2006). Structure and function of myosin filaments. Curr Opin Struct Biol.

[CR13] de Tombe PP, ter Keurs HE (1991). Sarcomere dynamics in cat cardiac trabeculae. Circ Res.

[CR14] Flashman E, Watkins H, Redwood C (2007). Localization of the binding site of the C-terminal domain of cardiac myosin-binding protein-C on the myosin rod. Biochem J.

[CR15] Geist J, Ward CW, Kontrogianni-Konstantopoulos A (2018). Structure before function: myosin binding protein-C slow is a structural protein with regulatory properties. FASEB J.

[CR16] Gonzalez-Sola M, Al-Khayat HA, Behra M, Kensler RW (2014). Zebrafish cardiac muscle thick filaments: isolation technique and three-dimensional structure. Biophys J.

[CR17] Gruen M, Gautel M (1999). Mutations in beta-myosin S2 that cause familial hypertrophic cardiomyopathy (FHC) abolish the interaction with the regulatory domain of myosin-binding protein-C. J Mol Biol.

[CR18] Gruen M, Prinz H, Gautel M (1999). cAPK-phosphorylation controls the interaction of the regulatory domain of cardiac myosin binding protein C with myosin-S2 in an on-off fashion. FEBS Lett.

[CR19] Harris SP (2021). Making waves: a proposed new role for myosin-binding protein C in regulating oscillatory contractions in vertebrate striated muscle. J Gen Physiol.

[CR20] Harris SP, Bartley CR, Hacker TA, McDonald KS, Douglas PS, Greaser ML, Powers PA, Moss RL (2002). Hypertrophic cardiomyopathy in cardiac myosin binding protein-C knockout mice. Circ Res.

[CR21] Harris SP, Belknap B, Van Sciver RE, White HD, Galkin VE (2016). C0 and C1 N-terminal Ig domains of myosin binding protein C exert different effects on thin filament activation. Proc Natl Acad Sci U S A.

[CR22] Harris SP, Lyons RG, Bezold KL (2011). In the thick of it: HCM-causing mutations in myosin binding proteins of the thick filament. Circ Res.

[CR23] Haselgrove JC (1975). X-ray evidence for conformational changes in the myosin filaments of vertebrate striated muscle. J Mol Biol.

[CR24] Heling L, Geeves MA, Kad NM (2020). MyBP-C: one protein to govern them all. J Muscle Res Cell Motil.

[CR25] Huang X, Li S, Gao S (2018). Exploring an optimal wavelet-based filter for cryo-ET imaging. Sci Rep.

[CR26] Irving M (2017). Regulation of contraction by the thick filaments in skeletal muscle. Biophys J.

[CR27] Jacques AM, Copeland O, Messer AE, Gallon CE, King K, McKenna WJ, Tsang VT, Marston SB (2008). Myosin binding protein C phosphorylation in normal, hypertrophic and failing human heart muscle. J Mol Cell Cardiol.

[CR28] Kampourakis T, Yan Z, Gautel M, Sun YB, Irving M (2014). Myosin binding protein-C activates thin filaments and inhibits thick filaments in heart muscle cells. Proc Natl Acad Sci U S A.

[CR29] Kensler RW (2005). The mammalian cardiac muscle thick filament: backbone contributions to meridional reflections. J Struct Biol.

[CR30] Kensler RW, Shaffer JF, Harris SP (2010). Binding of the N-terminal fragment C0–C2 of cardiac MyBP-C to cardiac F-actin. J Struct Biol.

[CR31] Koubassova NA, Tsaturyan AK, Bershitsky SY, Ferenczi MA, Padron R, Craig R (2022). Interacting-heads motif explains the X-ray diffraction pattern of relaxed vertebrate skeletal muscle. Biophys J.

[CR32] Kremer JR, Mastronarde DN, McIntosh JR (1996). Computer visualization of three-dimensional image data using IMOD. J Struct Biol.

[CR33] Lee K, Harris SP, Sadayappan S, Craig R (2015). Orientation of myosin binding protein C in the cardiac muscle sarcomere determined by domain-specific immuno-EM. J Mol Biol.

[CR34] Li A, Nelson S, Lee K, Previs S, Brack K, Previs M, Govindan S, Sadayappan S, Craig R, Warshaw D (2017). Skeletal myosin-binding protein C modulates actomyosin contractility in an isoform-dependent manner. Biophys J.

[CR35] Li X, Mooney P, Zheng S, Booth CR, Braunfeld MB, Gubbens S, Agard DA, Cheng Y (2013). Electron counting and beam-induced motion correction enable near-atomic-resolution single-particle cryo-EM. Nat Methods.

[CR36] Luther PK, Morris EP (2003). Cryoelectron microscopy of refrozen cryosections. J Struct Biol.

[CR37] Luther PK, Winkler H, Taylor K, Zoghbi ME, Craig R, Padron R, Squire JM, Liu J (2011). Direct visualization of myosin-binding protein C bridging myosin and actin filaments in intact muscle. Proc Natl Acad Sci U S A.

[CR38] Mahamid J, Pfeffer S, Schaffer M, Villa E, Danev R, Cuellar LK, Forster F, Hyman AA, Plitzko JM, Baumeister W (2016). Visualizing the molecular sociology at the HeLa cell nuclear periphery. Science.

[CR39] Marquez G, Pinto A, Alamo L, Baumann B, Ye F, Winkler H, Taylor K, Padron R (2014). A method for 3D-reconstruction of a muscle thick filament using the tilt series images of a single filament electron tomogram. J Struct Biol.

[CR40] Mastronarde DN (2005). Automated electron microscope tomography using robust prediction of specimen movements. J Struct Biol.

[CR41] McNamara JW, Li A, Dos Remedios CG, Cooke R (2015). The role of super-relaxed myosin in skeletal and cardiac muscle. Biophys Rev.

[CR42] Moos C (1981). Fluorescence microscope study of the binding of added C protein to skeletal muscle myofibrils. J Cell Biol.

[CR43] Moos C, Mason CM, Besterman JM, Feng IN, Dubin JH (1978). The binding of skeletal muscle C-protein to F-actin, and its relation to the interaction of actin with myosin subfragment-1. J Mol Biol.

[CR44] Mun JY, Previs MJ, Yu HY, Gulick J, Tobacman LS, Beck Previs S, Robbins J, Warshaw DM, Craig R (2014). Myosin-binding protein C displaces tropomyosin to activate cardiac thin filaments and governs their speed by an independent mechanism. Proc Natl Acad Sci U S A.

[CR45] Nag S, Trivedi DV, Sarkar SS, Adhikari AS, Sunitha MS, Sutton S, Ruppel KM, Spudich JA (2017). The myosin mesa and the basis of hypercontractility caused by hypertrophic cardiomyopathy mutations. Nat Struct Mol Biol.

[CR46] Oda T, Yanagisawa H (2020). Cryo-electron tomography of cardiac myofibrils reveals a 3D lattice spring within the Z-discs. Commun Biol.

[CR47] Offer G, Moos C, Starr R (1973). A new protein of the thick filaments of vertebrate skeletal myofibrils. Extractions, purification and characterization. J Mol Biol.

[CR48] Padron R, Alamo L, Craig R, Caputo C (1988). A method for quick-freezing live muscles at known instants during contraction with simultaneous recording of mechanical tension. J Microsc.

[CR49] Pettersen EF, Goddard TD, Huang CC, Couch GS, Greenblatt DM, Meng EC, Ferrin TE (2004). UCSF Chimera–a visualization system for exploratory research and analysis. J Comput Chem.

[CR50] Pfuhl M, Gautel M (2012). Structure, interactions and function of the N-terminus of cardiac myosin binding protein C (MyBP-C): who does what, with what, and to whom?. J Muscle Res Cell Motil.

[CR51] Ponnam S, Kampourakis T (2021). Microscale thermophoresis suggests a new model of regulation of cardiac myosin function via interaction with cardiac myosin-binding protein C. J Biol Chem.

[CR52] Previs MJ, Beck Previs S, Gulick J, Robbins J, Warshaw DM (2012). Molecular mechanics of cardiac myosin-binding protein C in native thick filaments. Science.

[CR53] Previs MJ, Prosser BL, Mun JY, Previs SB, Gulick J, Lee K, Robbins J, Craig R, Lederer WJ, Warshaw DM (2015). Myosin-binding protein C corrects an intrinsic inhomogeneity in cardiac excitation-contraction coupling. Sci Adv.

[CR54] Rahmanseresht S, Lee KH, O’Leary TS, McNamara JW, Sadayappan S, Robbins J, Warshaw DM, Craig R, Previs MJ (2021) The N terminus of myosin-binding protein C extends toward actin filaments in intact cardiac muscle. J Gen Physiol. 10.1085/jgp.202012726 10.1085/jgp.202012726 PMC7852460 33528507

[CR55] Razumova MV, Shaffer JF, Tu AY, Flint GV, Regnier M, Harris SP (2006). Effects of the N-terminal domains of myosin binding protein-C in an in vitro motility assay: evidence for long-lived cross-bridges. J Biol Chem.

[CR56] Reconditi M, Brunello E, Fusi L, Linari M, Martinez MF, Lombardi V, Irving M, Piazzesi G (2014). Sarcomere-length dependence of myosin filament structure in skeletal muscle fibres of the frog. J Physiol.

[CR57] Risi C, Belknap B, Forgacs-Lonart E, Harris SP, Schroder GF, White HD, Galkin VE (2018). N-terminal domains of cardiac myosin binding protein C cooperatively activate the thin filament. Structure.

[CR58] Shaffer JF, Kensler RW, Harris SP (2009). The myosin-binding protein C motif binds to F-actin in a phosphorylation-sensitive manner. J Biol Chem.

[CR59] Slot JW, Geuze HJ (1985). A new method of preparing gold probes for multiple-labeling cytochemistry. Eur J Cell Biol.

[CR60] Studer D, Humbel BM, Chiquet M (2008). Electron microscopy of high pressure frozen samples: bridging the gap between cellular ultrastructure and atomic resolution. Histochem Cell Biol.

[CR61] Tokuyasu KT (1973). A technique for ultracryotomy of cell suspensions and tissues. J Cell Biol.

[CR62] Tsukita S, Yano M (1985). Actomyosin structure in contracting muscle detected by rapid freezing. Nature.

[CR63] van Dijk SJ, Bezold Kooiker K, Mazzalupo S, Yang Y, Kostyukova AS, Mustacich DJ, Hoye ER, Stern JA, Kittleson MD, Harris SP (2016). The A31P missense mutation in cardiac myosin binding protein C alters protein structure but does not cause haploinsufficiency. Arch Biochem Biophys.

[CR64] Vijayakrishnan S, McElwee M, Loney C, Rixon F, Bhella D (2020). In situ structure of virus capsids within cell nuclei by correlative light and cryo-electron tomography. Sci Rep.

[CR65] Vydyanath A, Gurnett CA, Marston S, Luther PK (2012). Axial distribution of myosin binding protein-C is unaffected by mutations in human cardiac and skeletal muscle. J Muscle Res Cell Motil.

[CR66] Wang Z, Grange M, Wagner T, Kho AL, Gautel M, Raunser S (2021). The molecular basis for sarcomere organization in vertebrate skeletal muscle. Cell.

[CR67] Watkins H, Conner D, Thierfelder L, Jarcho JA, MacRae C, McKenna WJ, Maron BJ, Seidman JG, Seidman CE (1995). Mutations in the cardiac myosin binding protein-C gene on chromosome 11 cause familial hypertrophic cardiomyopathy. Nat Genet.

[CR68] Weith AE, Previs MJ, Hoeprich GJ, Previs SB, Gulick J, Robbins J, Warshaw DM (2012). The extent of cardiac myosin binding protein-C phosphorylation modulates actomyosin function in a graded manner. J Muscle Res Cell Motil.

[CR69] Wendt T, Taylor D, Trybus KM, Taylor K (2001). Three-dimensional image reconstruction of dephosphorylated smooth muscle heavy meromyosin reveals asymmetry in the interaction between myosin heads and placement of subfragment 2. Proc Natl Acad Sci U S A.

[CR70] Whitten AE, Jeffries CM, Harris SP, Trewhella J (2008). Cardiac myosin-binding protein C decorates F-actin: implications for cardiac function. Proc Natl Acad Sci U S A.

[CR71] Woodhead JL, Zhao FQ, Craig R, Egelman EH, Alamo L, Padron R (2005). Atomic model of a myosin filament in the relaxed state. Nature.

[CR72] Xiong Q, Morphew MK, Schwartz CL, Hoenger AH, Mastronarde DN (2009). CTF determination and correction for low dose tomographic tilt series. J Struct Biol.

[CR73] Yang S, Tiwari P, Lee KH, Sato O, Ikebe M, Padron R, Craig R (2020). Cryo-EM structure of the inhibited (10S) form of myosin II. Nature.

[CR74] Zhang K (2016). Gctf: Real-time CTF determination and correction. J Struct Biol.

[CR75] Zoghbi ME, Woodhead JL, Moss RL, Craig R (2008). Three-dimensional structure of vertebrate cardiac muscle myosin filaments. Proc Natl Acad Sci U S A.

