# Supplementary material for: Cryo-electron tomography of intact cardiac muscle reveals myosin binding protein-C linking myosin and actin filaments
Source: J Muscle Res Cell Motil. 2023 Apr 28;44(3):165–78. doi: 10.1007/s10974-023-09647-3 (PMC10542292; doi:10.1007/s10974-023-09647-3)
Supplement: Supplementary file 1 — Supplementary file1 (DOCX 3622 KB) [file 10974_2023_9647_MOESM1_ESM.docx]

Cryo‑electron tomography of intact cardiac muscle reveals myosin

binding protein‑C linking myosin and actin filaments

Xinrui Huang · Iratxe Torre · Michele Chiappi · Zhan Yin · Anupama Vydyanath · Shuangyi Cao ·

Oliver Raschdorf · Morgan Beeby · Bonnie Quigley · Pieter P. de Tombe · Jun Liu · Edward P. Morris ·

Pradeep K. Luther

**Supplementary Information**

**Preparation of samples shown in Figure S9**

(a) Rat heart – this study. (b) Rabbit heart was harvested from a sacrificed animal as described previously (Thirlwell et al., 1994) and cryosections prepared from papillary muscles. (c) Human cardiac muscle was obtained following myectomy operation as described previously (Vydyanath et al., 2012). (d) Pig heart was obtained from an abattoir. (e) Cat heart tissue was obtained as described previously (van Dijk et al., 2016). (f) Skeletal tibialis anterior muscle was obtained from a mouse with MLP-ko genotype; we do not expect it to have a skeletal phenotype (Gehmlich et al., 2010). (g) Frog skeletal sartorius muscle was fast-frozen/freeze-substituted/resin embedded as described previously ( (Luther et al., 2011). Cryosections were cut with an RMC ultramicrotome MT7 fitted with CR20 cryo-attachment (rabbit, human, pig, skeletal mouse) or Leica UC6 ultramicrotome fitted with a Leica FC6 cryochamber (rat and cat). The cryosections were negative stained with 2% ammonium molybdate.

**Supplementary Information Figure and Captions**


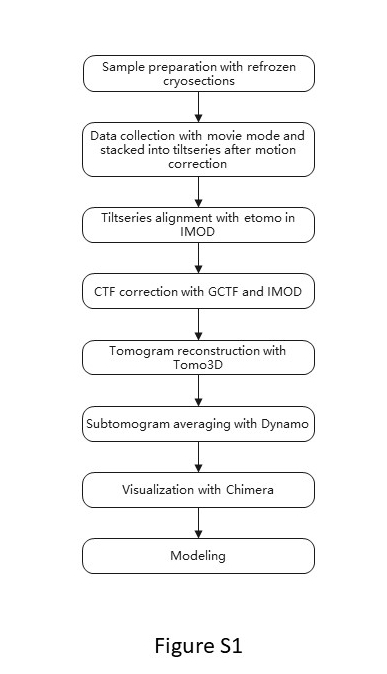


**Figure S1**

Flow diagram of the steps used for this study.


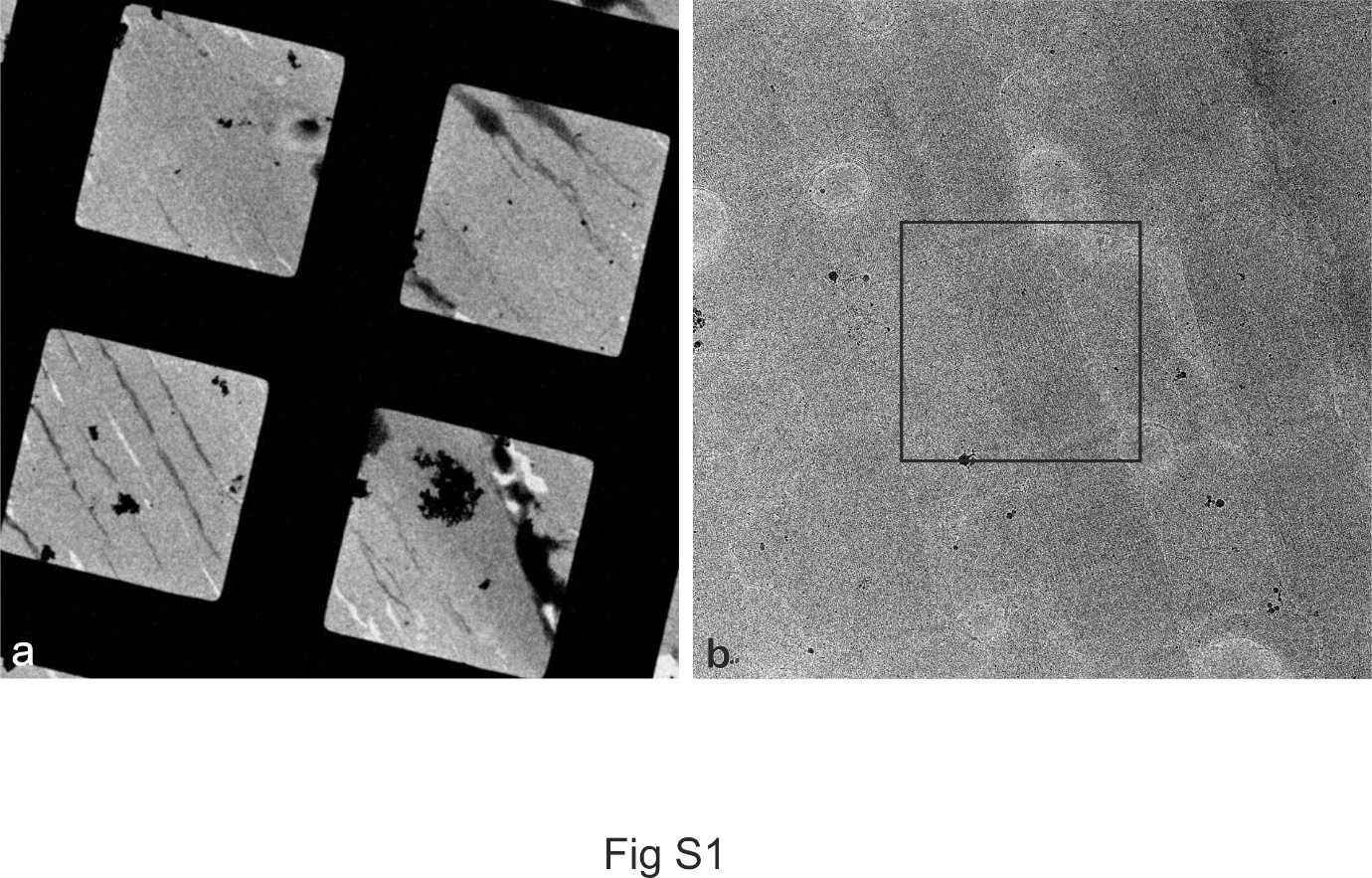


**Figure S2**

Preparatory steps for cryo-em of muscle cryosections. (a) Survey picture from grid atlas of frozen cryosections showing myofibrils running diagonally to the left. (b) Higher magnification view used for selecting tomography target regions showing an example of the final area used, approximately ~ 2µm square, close to the sarcomere length of 2.2 µm used in this study.


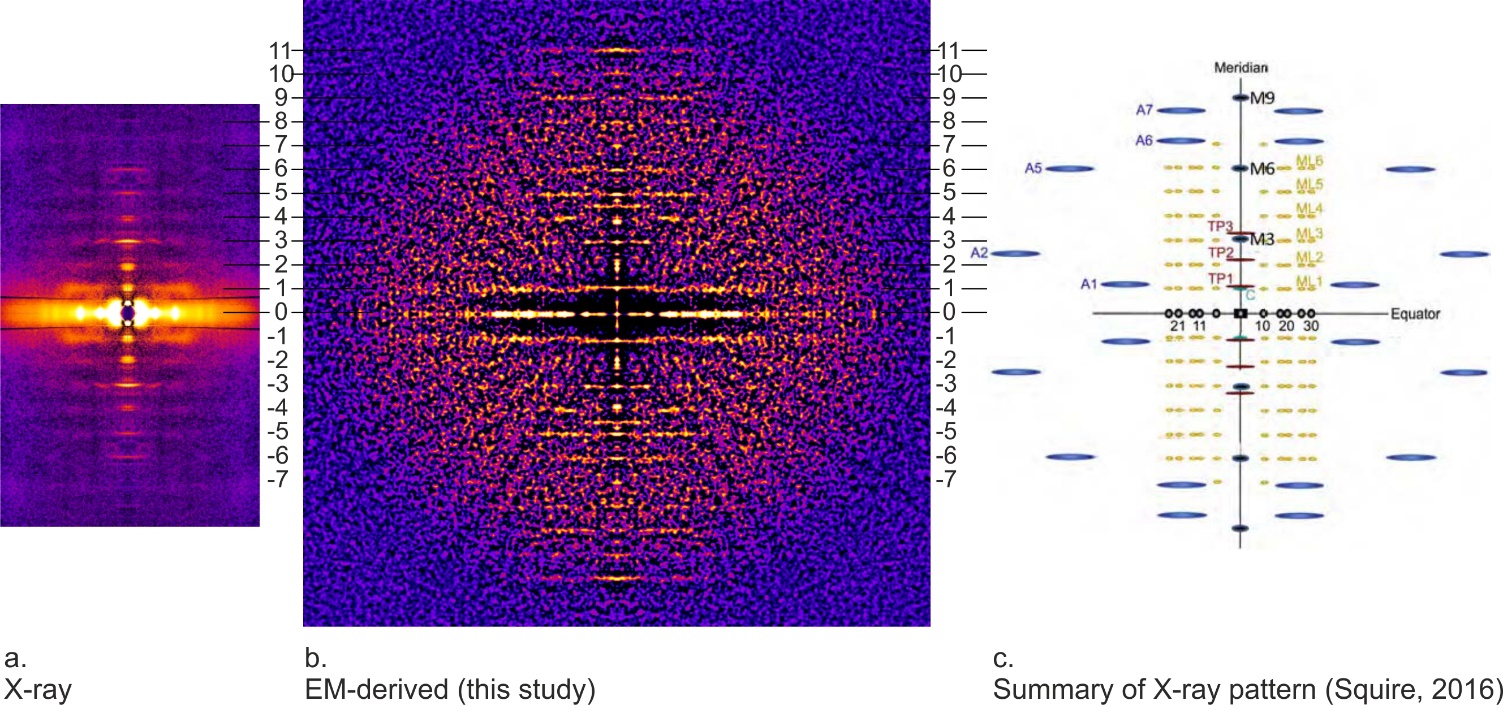


**Figure S3**

Demonstration of the exquisite preservation of fine structure in the refrozen Tokuyasu cryosection of cardiac muscle used in this study. We compare the mean Fourier transform calculated from the Fourier transforms of the 10 tomogram projections used in this study (b) with the X-ray diffraction pattern of rat cardiac trabecula (a) and schematic of the X-ray pattern (c) showing the origin of the reflections from myosin (light green) and actin (blue). The X-ray pattern is dominated by layer-lines of spacing ~430Å, resulting from the near helical arrangement of myosin crossbridges. All 3 figures are at the same scale; the layer-lines, indicated by the set of lines are numbered 0 to 11, depict orders of 430 Å. The extent of the layer-lines in the mean EM-derived Fourier transform and the close match of the Fourier transform with the X-ray pattern of live muscle demonstrates there is excellent preservation of fine structure in our samples.

(a) X-ray pattern modified from Ait-Mou et al (Ait-Mou et al., 2016), courtesy of Pieter de Tombe, (c) schematic diagram from (Squire, 2016), courtesy of Carlo Knupp.


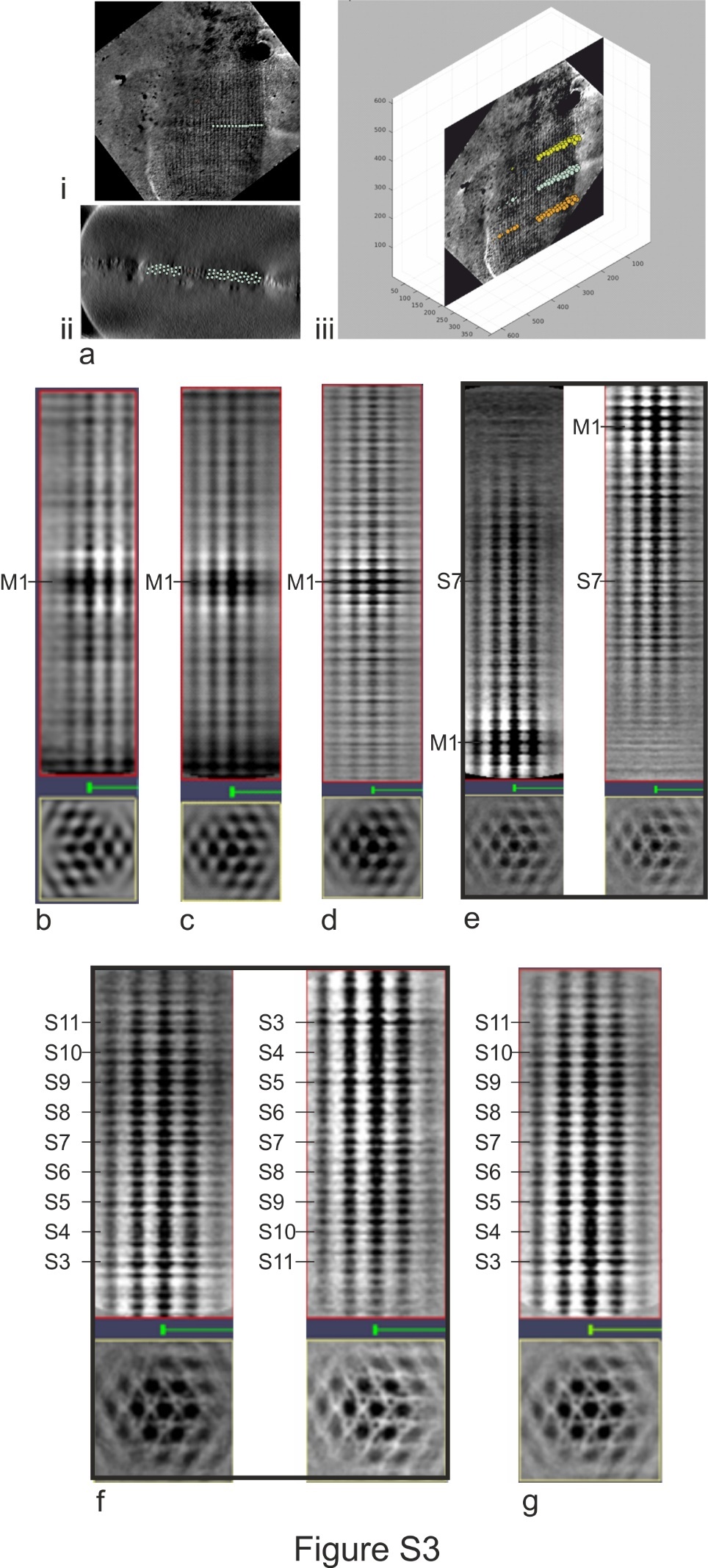


**Figure S4**

Stepwise subtomogram averaging (STA) of cardiac muscle cryosection tomograms using Dynamo (Castano-Diez et al, 2012). All tomograms were rotated to make filament axes along Z. Thick filaments positions were marked in side-on view and in cross-section at the distinct M1 stripe at the centre of the M-band (a). (b) Selected template centred at M1 constructed by STA from only one tomogram; 6x binned. (c) STA of all 10 selected tomograms centred at M1; 6x binned. (d) STA centred at M1; 4x binned. (e) STA for each ~half A-band (up and down) centred at stripe S7; 4x binned. (f) STA for the stripe regions 1-11 in the Up and Down A-bands; 4x binned. (g) STA after combining the Up and Down regions; 4x binned. Further STA was done using smaller boxes centred at individual stripes for 2x binned and finally unbinned regions.


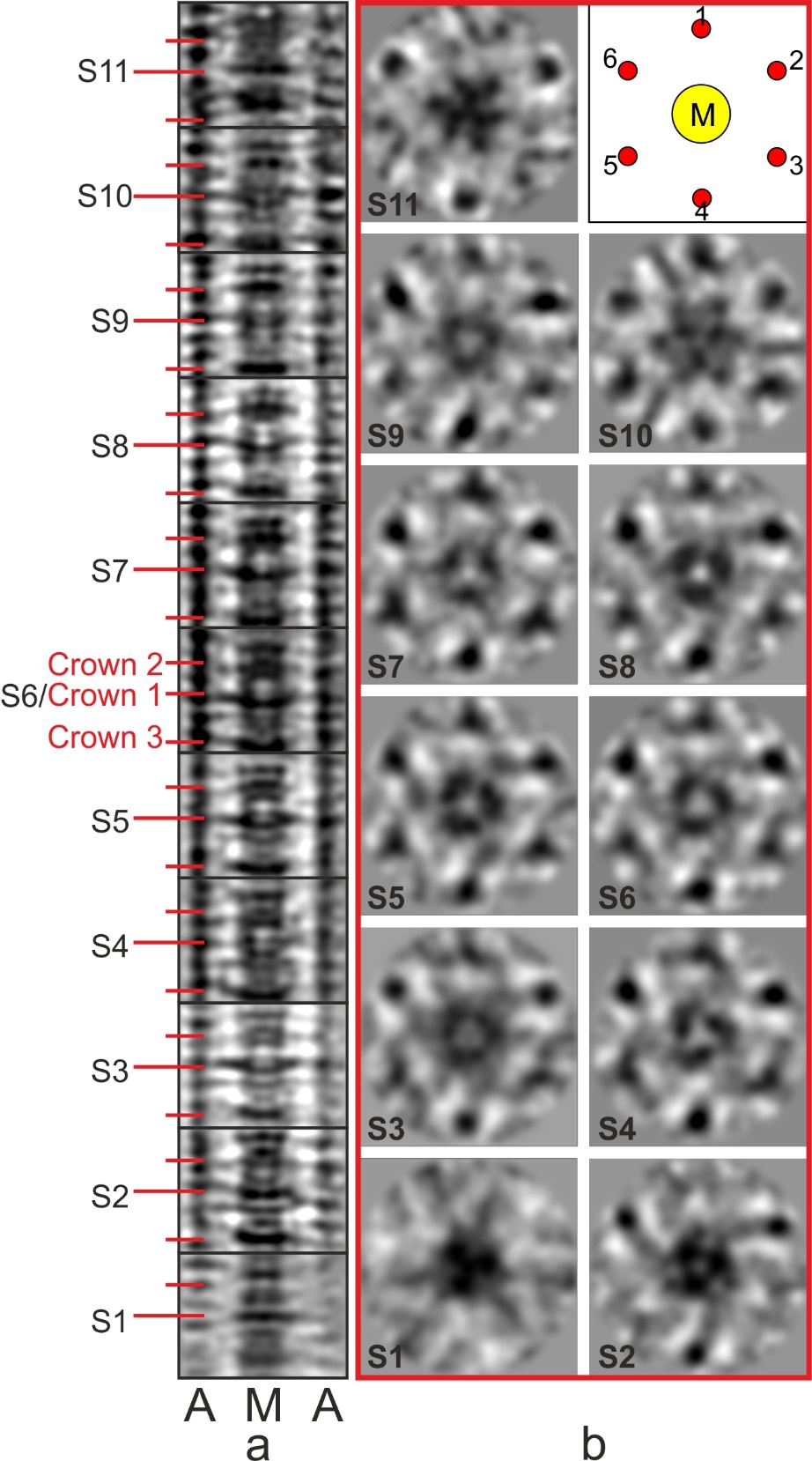


**Figure S5**

Grey level version of Fig 3 showing subtomogram average of 430 Å regions centred at individual MyBP-C stripes with black representing protein. Each image depicts a projection of a central slice, ~50 Å thick, of the respective tomogram. (a) Side-on view of a composite thick filament (M) between 2 actin filaments (A), generated by stacking axial 430 Å segments centred at respective stripe. (b) Cross-sections views at each stripe.


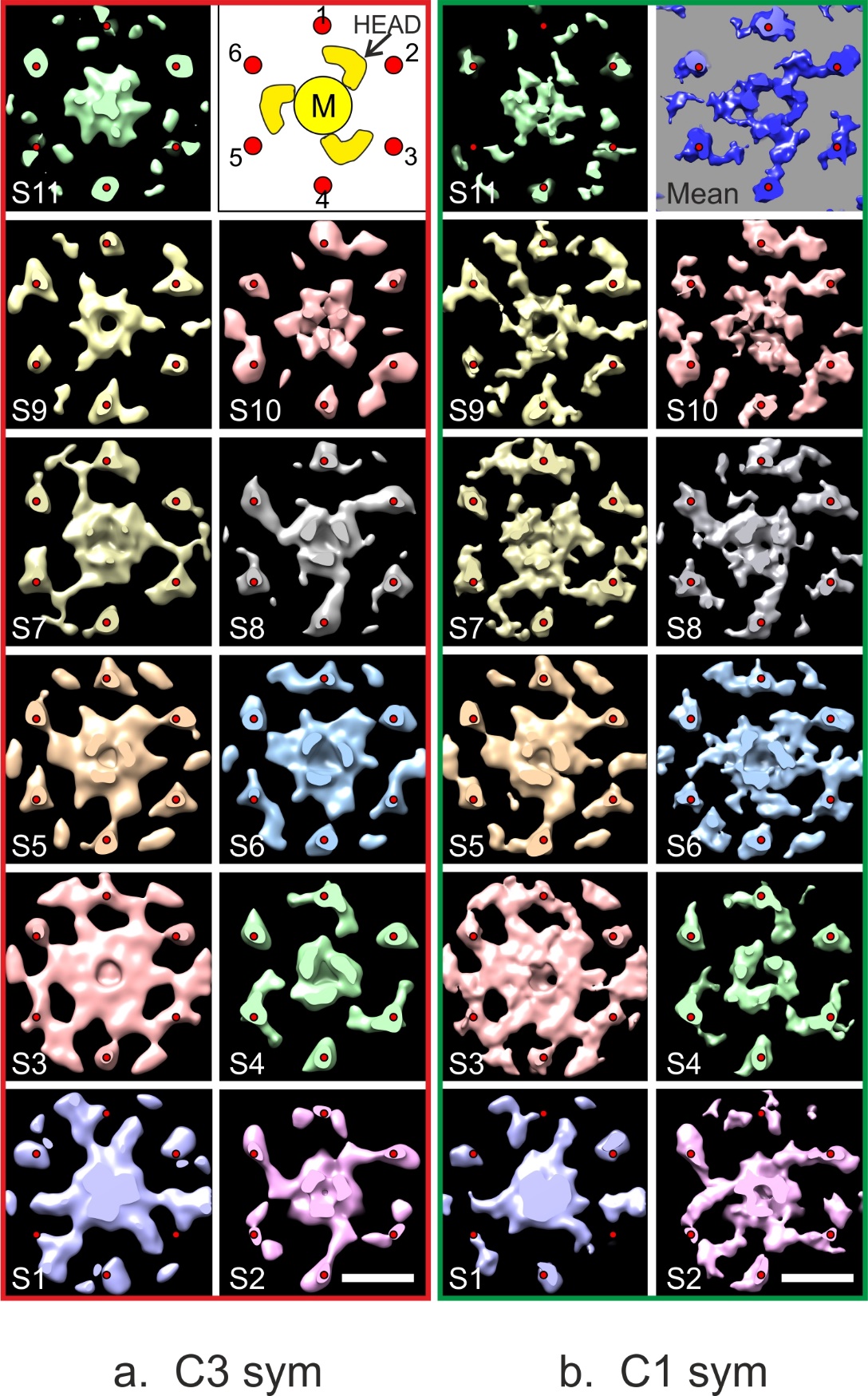


**Figure S6**

Fine structure of all MyBP-C stripes with C3 symmetry imposed (a) and C1 symmetry (b). The C3 panel is repeated from Fig 3 for ease of comparison here. The contour levels in Chimera were 0.4 and 0.5 respectively for C3 and C1 symmetry views except S3 panels, which were higher, 0.5 and 0.6, as the myosin filament backbone was too large otherwise. In the centre of each image is a myosin filament cross-section surrounded by 6 actins, marked with red circles in bottom row. The top right panel shows the mean structure of the C-zone stripes with C1 symmetry. At each MyBP-C stripe, there are about 2 dissimilar links to actin formed by MyBP-C. Since there are 3 MyBP-C at each stripe, the non-recovery of the 3^rd^ MyBP-C probably means that it is more dynamic, hence is smeared out. Scale bar = 200 Å.


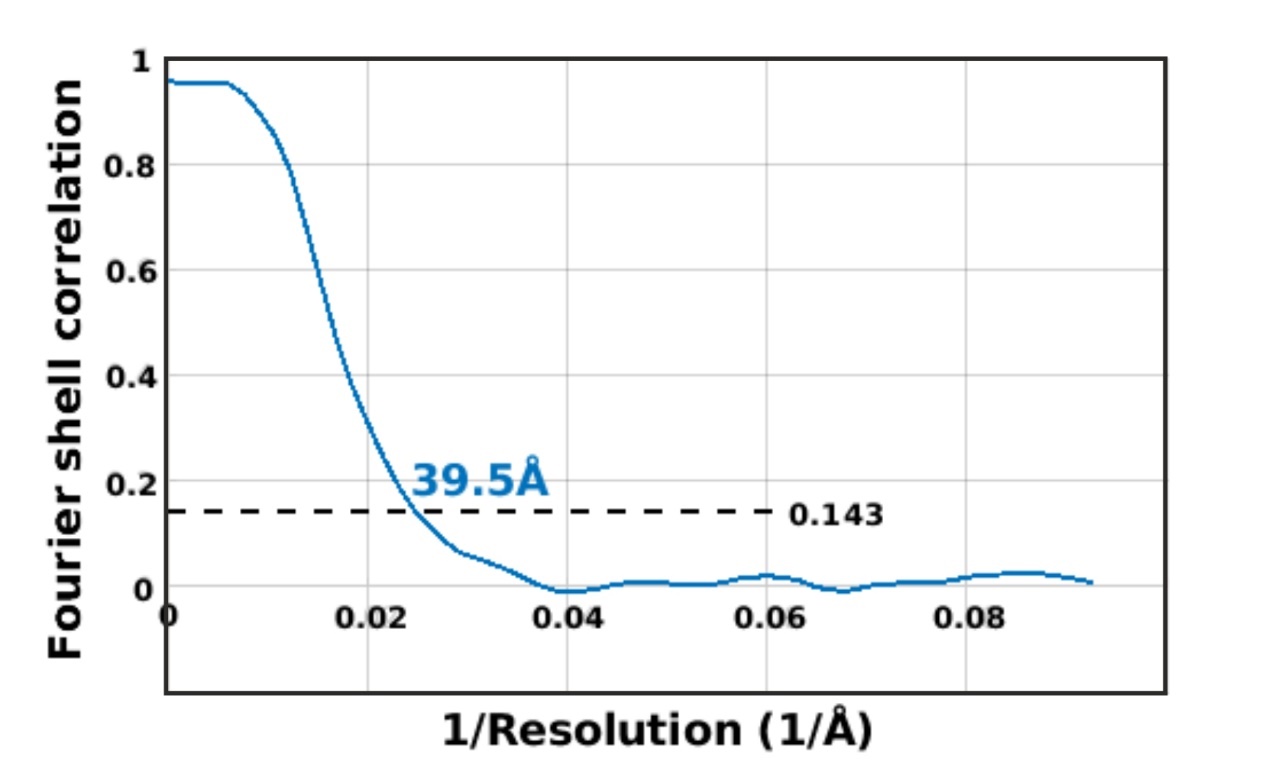


**Figure S7**

Resolution of mean 430 Å MyBP-C period. We estimate the resolution to be 39.5 Å using the 0.143 criterion.


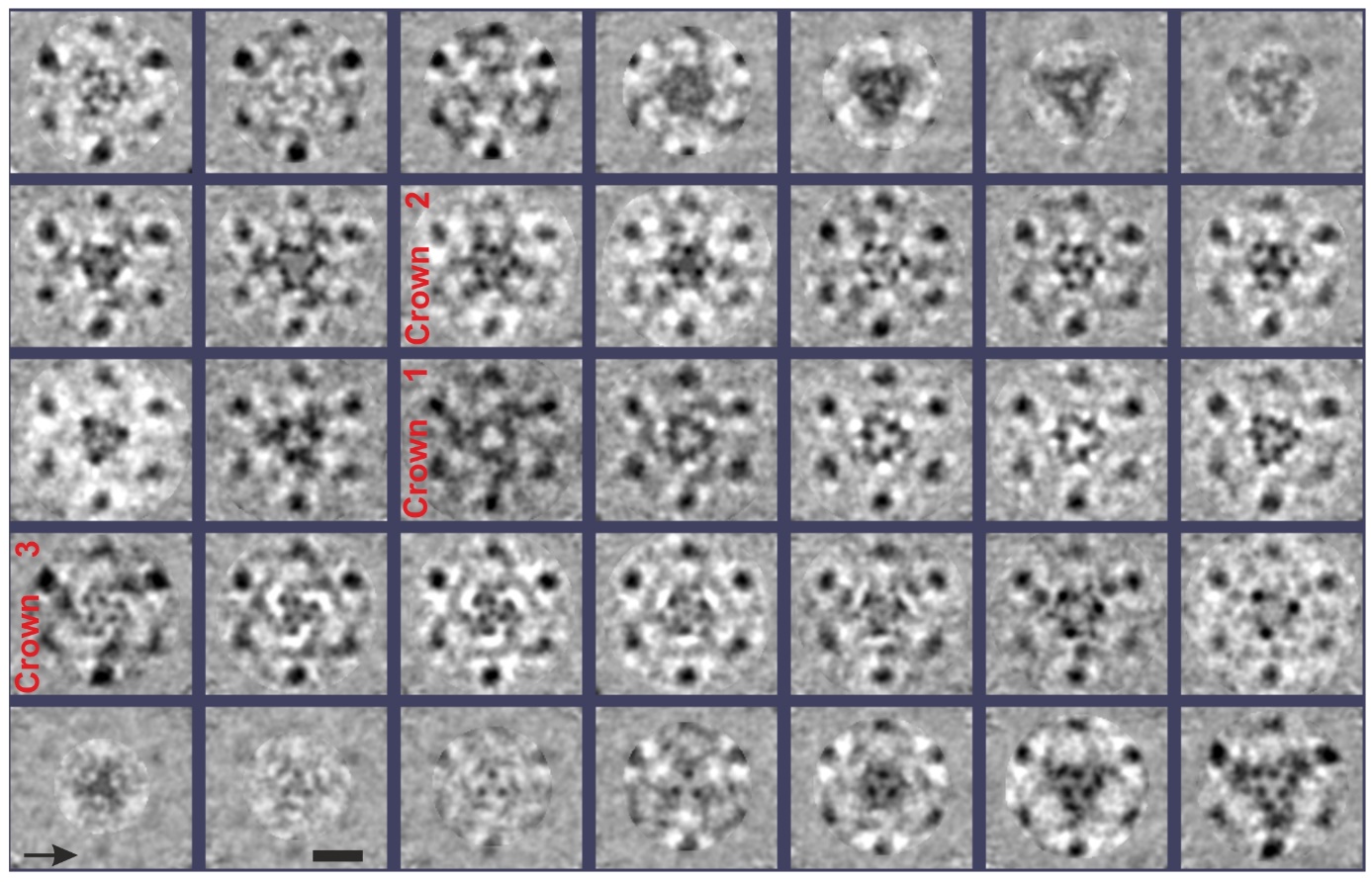


**Figure S8**

Thin ~19 Å slices through the averaged C-zone tomogram shown in Fig 4 depicting density in grey-level shading (black is protein). The order of the slices is rightwards from bottom left (arrow) followed by rightward traverse of each upper row. Each slice has a myosin filament at the centre surrounded by 6 actins. The locations for Crowns 3, 1 and 2 are marked (Crown 1 etc). At Crown 1 location there is prominent linking density between myosin and actin which we ascribe to cMyBP-C. Scale bar = 200 Å.


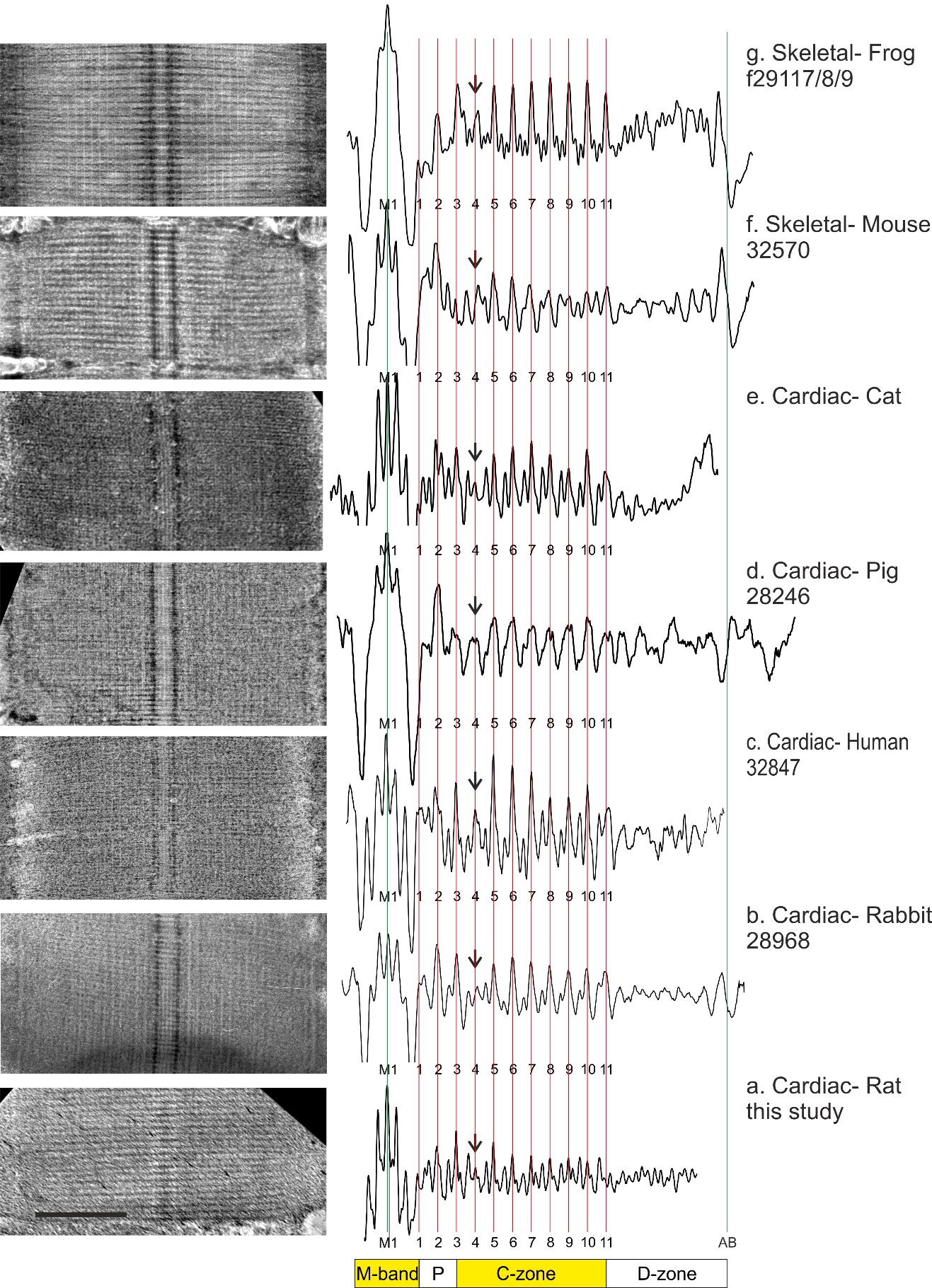


**Figure S9**

Making the case that Stripe 4 MyBP-C is different from MyBP-C at other stripes. Analysis performed on cryosections (a-f) and a resin section (g) of mammalian heart muscles (a-e) and skeletal muscle of mouse (f) and frog (g). Left panel, EM images of A-bands in each muscle. The cryosections are negative-stained (a-f) hence images are depicted with protein density as light. In the right panel, showing the mean plot profiles, the 2 vertical green lines mark the centre of the M-band, M1, and the edge of the A-band (AB) and the red lines mark the 11 430Å spaced stripes due to accessory proteins in the A-band. The plot profiles show that the density is distinctly weaker at Stripe 4 (arrows). We propose that the low density at Stripe 4 may be due to MyBP-C running axially along the thick filament in contrast to the radial arrangement at other stripes. Fig S9c, Modified from Vydyanath et al (2012); Fig S9g, modified from Luther et al (2011). Scale bar = 0.5 µm.

**Movie Captions**

**Movie 1**. Example of a combined tilt series of a refrozen cryosection of rat cardiac muscle. Gold particles and some ice contamination can be seen on the surfaces.

**Movie 2**. Paging through tomogram slices of tilt series in Movie 1. The movie starts from the centre, straddles to one side and through to the other which is labelled with gold particles. Two sarcomeres are present running diagonally to the upper right with clear thick filaments and M-bands in the centre and Z-bands near the corners.

**Movie 3a.** Illustrating the special nature of the 3D structure of MyBP-C Stripe 4. At start, the end-on view shows a multitude of links between myosin and actin which are resolved upon transition to side-on view as densities at different 3D levels. The stripe level does not have strong myosin-actin density like the other MyBP-C stripes like the examples shown for Stripes 5 and 8 (Movies 3b and 3c).

**Movies 3b and 3c.** Movies showing examples of the 3D structure of individual MyBP-C 430 Å repeats at Stripes 5 and 8, respectively. At the start of the movie, the end-on views show MyBP-C making clear links to actin. The side-on views show MyBP-C density and the density due to the heads at crowns 1-3.

**Movie 4**. Illustrating the special nature of Stripe 4 by comparing cardiac reconstruction (this study) with frog skeletal reconstruction (Luther et al., 2011). The structure at different time points follows. (start) Side-on view of skeletal reconstruction showing Stripe 3 to Stripe 5 regions. Stripe 4 lacks and Stripe 3 and 5 have protruding radial MyBP-C density. (10s) Brief overlay with individual cardiac Stripe 3, 4 and 5 averaged tomograms. (16s) Cardiac Stripe 3, 4 and 5 tomograms. There is much less myosin-actin density at Stripe 4 than 3 and 5. (25s) Overlay of skeletal and cardiac. (37s) Skeletal filament tilted forward to show radial actin bound density at Stripe 3 and 5. (46s) Overlay of tilted skeletal and cardiac reconstructions (55s) Cardiac reconstruction tilt view, showing pancake-like density at Stripe 3 and 5 compared with lack of density at Stripe 4.

**Table S1**. Number of filaments (particles) contributing to the subtomogram averages shown.

| Procedure | Number of filaments (particles) |
| --- | --- |
| Fig S4, (b-c): M-band, 6x Binned  (d-g) 4x Binned M-band Bin4, A-band Bin4U and A-band Bin4D, | 1031 |
| Fig S4g A-band, combined Up and Down half sarcomeres. | 2062 |
| Number of Subtomograms used in Fig 3: |  |
| S3 (i.e. stripe 3) | 1966 |
| S4 | 1937 |
| S5 | 1944 |
| S6 | 1926 |
| S7 | 1916 |
| S8 | 1863 |
| S9 | 1858 |
| S10 | 1805 |
| S11 | 1585 |
| Thick 3 to 11 | 15099 (number of MyBP-C 430 Å repeats) |
| 2 classes:  Class 1  Class 2 | 7681  7418 |
| 278 for the merged map (firstC2Ref1Ex0.035.mrc) after alignment for class 1 and setting CC threshold=0.035, Fig 4. | 7681 (278) |

**References for Supplementary Information**

Ait-Mou, Y., K. Hsu, G.P. Farman, M. Kumar, M.L. Greaser, T.C. Irving, and P.P. de Tombe. 2016. Titin strain contributes to the Frank-Starling law of the heart by structural rearrangements of both thin- and thick-filament proteins. *Proc Natl Acad Sci U S A*. 113:2306-2311.

Castano-Diez D, Kudryashev M, Arheit M, Stahlberg H. 2012. Dynamo: a flexible, user-friendly development tool for subtomogram averaging of cryo-EM data in high-performance computing environments. J Struct Biol 178:139–151

Gehmlich, K., E. Ehler, A. Perrot, D.O. Furst, and C. Geier. 2010. MLP: A Stress Sensor Goes Nuclear" by Sylvia Gunkel, Jorg Heineke, Denise Hilfiker-Kleiner, Ralph Knoll, J Mol Cell Cardiol. 2009;47(4):423-5. *J Mol Cell Cardiol*. 48:424-425; author reply 426-427.

Luther, P.K., H. Winkler, K. Taylor, M.E. Zoghbi, R. Craig, R. Padron, J.M. Squire, and J. Liu. 2011. Direct visualization of myosin-binding protein C bridging myosin and actin filaments in intact muscle. *Proc Natl Acad Sci U S A*. 108:11423-11428.

Squire, J.M. 2016. Muscle contraction: Sliding filament history, sarcomere dynamics and the two Huxleys. Glob Cardiol Sci Pract. 2016 Jun 30;2016(2):e201611. doi: 10.21542/gcsp.2016.11.

Thirlwell, H., J.E. Corrie, G.P. Reid, D.R. Trentham, and M.A. Ferenczi. 1994. Kinetics of relaxation from rigor of permeabilized fast-twitch skeletal fibers from the rabbit using a novel caged ATP and apyrase. *Biophys J*. 67:2436-2447.

van Dijk, S.J., K. Bezold Kooiker, S. Mazzalupo, Y. Yang, A.S. Kostyukova, D.J. Mustacich, E.R. Hoye, J.A. Stern, M.D. Kittleson, and S.P. Harris. 2016. The A31P missense mutation in cardiac myosin binding protein C alters protein structure but does not cause haploinsufficiency. *Arch Biochem Biophys*. 601:133-140.

Vydyanath, A., C.A. Gurnett, S. Marston, and P.K. Luther. 2012. Axial distribution of myosin binding protein-C is unaffected by mutations in human cardiac and skeletal muscle. *J Muscle Res Cell Motil*. 33:61-74.
